# Effects of attenuated influenza virus administration to young children on pneumococcal density and acquisition in UK families: a randomised controlled trial

**DOI:** 10.1016/j.lanmic.2026.101422

**Published:** 2026-10

**Authors:** Jane Metz, George Qian, Begonia Morales-Aza, Jennifer Oliver, Elizabeth Oliver, Helen Rice, Kaltun Duale, Paul Heath, Saul Faust, Matthew Snape, Stephen Hughes, Laura Hole, Rebecca Mann, Fiona Shackley, Peter Rudd, Siân Ludman, Bradford Gessner, Leon Danon, Adam Finn

**Affiliations:** a Bristol Vaccine Centre, School of Cellular and Molecular Medicine and School of Population Health Sciences, University of Bristol, Bristol, UK; b Department of Engineering Mathematics, University of Bristol, Bristol, UK; c Vaccine Institute, Centre for Neonatal and Paediatric Infection, City St Georges, University of London, London, UK; d NIHR Southampton Clinical Research Facility, University of Southampton, Southampton, UK; e Oxford Vaccine Group, Department of Paediatrics, University of Oxford, Oxford, UK; f Manchester University NHS Foundation Trust, Department of Paediatric Allergy and Immunology, Royal Manchester Children’s Hospital, Manchester, UK; g Department of Paediatrics, Gloucestershire Royal Hospital, Gloucester, UK; h Department of Paediatrics, Musgrove Park Hospital, Taunton, UK; i Department of Paediatrics, Sheffield Children’s Hospital, Sheffield, UK; j Department of Paediatrics, Royal United Hospital Bath NHS Foundation Trust, Bath, UK; k Department of Paediatrics, Royal Devon and Exeter NHS Foundation Trust, Exeter, UK; l Pfizer Vaccines, Collegeville, PA, USA; m Bristol Royal Hospital for Children, University Hospitals Bristol and Weston NHS Foundation Trust, Bristol, UK

## Abstract

**Background:**

The live attenuated influenza vaccine (LAIV) is offered in the UK to young children, protecting against influenza for those vaccinated, and as an indirect protection for the wider community. LAIV has also been shown to increase carriage density of *Streptococcus pneumoniae*, to an extent, in children. This study sought to confirm whether the vaccine, as a viral proxy, leads to an increase in carriage density in children and, if so, whether this increase augments *S pneumoniae* acquisition in household contacts.

**Methods:**

We conducted a randomised control study (ISRCTN10720581, now complete) across ten UK sites. We included families having a child aged 2 years (index child) and due to receive their first LAIV dose under National Health Service criteria, and at least two other household contacts. Participants were excluded when the index child was ineligible for LAIV, had primary or secondary immunodeficiency, or was currently enrolled in other trials or circumstances that could jeopardise participation or data integrity during the study. Families were randomly assigned (1:1) to either the early LAIV group or late LAIV group using a single central allocation service with computerised randomisation for all sites. After randomisation, families and clinical research staff were unmasked; however, laboratory and analytical research staff remained masked. Nasopharyngeal swabs were collected from all participants over five visits, each 2 weeks apart, and tested for pneumococcal carriage by quantitative PCR for *lytA*, with a cycle threshold (Ct) cutoff of 35, and Ct values converted to gene copies per mL. LAIV was given to index children at visit 1 in the early LAIV group and at visit 3 in the late LAIV group. The coprimary outcomes of this study were *S pneumoniae* density in index children at 14 and 28 days after LAIV as compared with that on day 0, and the prevalence, at days 14 and 28, of *S pneumoniae* colonisation in the household contacts of index children who were *S pneumoniae*-positive at baseline. We developed regression models to analyse the association between vaccination and whether an increase in pneumococcal density was observed 14 and 28 days later, in addition to an increase in the odds of *S pneumoniae* acquisition in household members after administering LAIV. Serious adverse events were recorded if reported to the study team and were assessed by the Chief Investigator for seriousness, expectedness, and relatedness to LAIV or study procedures.

**Findings:**

405 families were enrolled between Oct 3, and Oct 30, 2017, and between Sept 27, and Nov 2, 2018. 205 families (703 participants) were allocated to the early LAIV group and 200 (676 participants) to the late LAIV group. Data on sex were available for 402 of 405 index participants. In the early LAIV group, 116 (58%) of 202 index children were female and 86 (42%) were male, and the median age was 2·47 years (IQR 0·331); in the late LAIV group, 91 (46%) of 200 index children were female and 108 (54%) were male, and the median age was 2·47 years (0·333). In the early LAIV group, 276 (59%) of 470 household contacts were female and 194 (41%) were male, and the median age of contacts was 33·8 (IQR 31·7); in the late LAIV group 272 (60%) of 453 household contacts were female and 181 (40%) were male, and the median age of contacts was 33·1 (IQR 31·4). 388 (96%) of 405 households, including 1309 (95%) of the 1379 participants, completed the study. Regression analyses showed that there was a 2·5-times (95% CI 1·5–4·3, p=0·0008) increase in the odds of vaccinated children having increased pneumococcal density 14 days later, compared with that in unvaccinated children, and a 2·0-times (95% CI 1·2–3·5, p=0·015) increase in the odds of household contacts *S pneumoniae* acquisition. These effects appear to have attenuated by day 28, on which there was a 1·5 times (95% CI 0·88-2·5, p=0·14) increase in odds of vaccinated children having increased pneumococcal density compared with that in unvaccinated children and a 0·89 times (95% CI 0·53–1·5, p=0·67) the odds of household contacts *S pneumoniae* acquisition.

Three serious adverse events related to pregnancy and breastfeeding were reported, which were deemed to be unrelated to the study.

**Interpretation:**

Our results show that in young children colonised with *S pneumoniae*, infection with an attenuated influenza virus in the 2 weeks after vaccination increases the risk of pneumococcal acquisition among their close contacts. This effect appears to be driven by a vaccine-induced rise in bacterial density. On the basis of our results, density of bacterial nasal colonisation in young children could be used to model transmission dynamics, both at the individual and population levels.

**Funding:**

University of Bristol, Gates Foundation, Pfizer, European Society of Paediatric Infectious Diseases.

## Introduction

Despite the global disease burden of *Streptococcus pneumoniae*, factors underlying *S pneumoniae* transmission have not been fully evaluated. Substantial evidence supports that upper respiratory tract pneumococcal colonisation, although a prerequisite for disease on rare occasions, is not of itself a cause of ill health. However, upper respiratory tract pneumococcal colonisation does sustain person-to-person transmission^1^^,^^2^ and, thus, any detectable change in colonisation density could affect epidemiology at the population level. The relationship between colonisation density and disease is also unknown, although some association data have been published, indicating that increased carriage density increases the risk of translocation into the lower respiratory tract^3^ and that intercurrent respiratory viral infections could also promote invasion and inflammation by other means.^4^ Young children are most likely the main reservoir and transmission drivers of *S pneumoniae*—they carry *S pneumoniae* at the highest rates, particularly when attending daycare; carers of young children carry *S pneumoniae* at higher rates than non-carer adults of similar age; and high vaccination coverage against *S pneumoniae* in early childhood with pneumococcal conjugate vaccines reduces vaccine-type disease and carriage in the whole population.^1^^,^^2^

**Research in context**

**Evidence before this study**

There is growing evidence of association between pneumococcal carriage density and respiratory viral coinfection, including influenza A. Two human randomised control studies have shown that the nasal live attenuated influenza vaccine (LAIV) given to young children increases pneumococcal carriage density. The effects of viral respiratory tract infections or pneumococcal carriage density, or both, on pneumococcal transmission have not been studied in humans. We searched PubMed for original research articles published in English from database inception up until April 30, 2024, exploring the effects of viral upper respiratory tract infections and LAIV on pneumococcal carriage, density, acquisition, clearance, and transmission, using the following terms in combination: “*Streptococcus pneumoniae*”, “pneumococcus”, “carriage”, “colonisation”, “viral respiratory tract infection”, “influenza”, “Live attenuated influenza vaccine”, “density”, “acquisition”, “clearance”, and “transmission”. We included studies in humans of all ages and that reported on pneumococcal transmission as an outcome. There were no studies assessing pneumococcal transmission in humans after LAIV.

**Added value of this study**

This randomised controlled study confirms that the tetravalent LAIV increases pneumococcal carriage density in recipients and shows increased acquisition in their household contacts compared with that in family members of unvaccinated controls. Post-hoc observational analysis showed that acquisition of *Streptococcus pneumoniae* in household contacts was more common when children were high-density carriers. This study, for the first time, shows that LAIV and increased carriage density are associated with increased pneumococcal acquisition in close household contacts.

**Implications of all the available evidence**

Factors that increase pneumococcal carriage density, such as viral respiratory tract infections, might contribute to increased pneumococcal transmission. Thus, measures to curb respiratory viral infections could play a key role in preventing pneumococcal disease.

There have been few studies evaluating household *S pneumoniae* transmission and, to our best knowledge, none investigating the effect of *S pneumoniae* density on household transmission. The intranasal live attenuated influenza vaccine (LAIV) is offered every autumn in the UK childhood immunisation programme to children aged 2 years and older. LAIV provides effective protection against influenza for those vaccinated and indirect protection for the wider community.^5^ LAIV has also been shown to increase *S pneumoniae* carriage density in mice and, to some degree, in children.^6^, ^7^, ^8^ This attenuated upper respiratory tract infection can, therefore, be used to investigate whether increases in *S pneumoniae* density in carriers augment acquisition in their contacts. We conducted a randomised controlled trial to assess the effect of LAIV administration on *S pneumoniae* carriage density in children aged 2 years who were previously LAIV-naive and on onward acquisition of *S pneumoniae* in their household contacts.

## Methods

### Study design and participants

This multisite, randomised controlled trial study recruited healthy boys and girls whose second birthday fell between Jan 1, and Aug 31, of the recruitment year and who were aged at least 2 years on their first study visit and eligible to receive their first LAIV dose. These children were residents near the recruiting centres, fulfilled eligibility criteria after a health check, and had parent(s) or legal guardians who provided signed informed consent; only children with at least two other household residents willing to participate were enrolled. Family contacts of the index children, who consented or assented when appropriate, also participated by completing procedures and providing nasopharyngeal samples. Exclusion criteria for all participants included the index child being ineligible for LAIV, primary or secondary immunodeficiency, and current enrolment in other trials or circumstances that could jeopardise participation or data integrity during the study. Temporary exclusion criteria for nasal spray administration to the index child included fever, recent immunisation (within 3 days for non-live and 21 days for any live vaccine) or plan to administer another vaccine within 7 days of study, active wheezing or asthma symptoms, blocked nose, or other health issues that could interfere with safe and effective participation; additionally, initiating long-term immunosuppressant or immune-modifying treatments during the study resulted in exclusion. The full inclusion and exclusion criteria are listed in the appendix (pp 10–11).

43 544 letters inviting study participation were sent to eligible households within the vicinity of the ten study sites—Bristol, Oxford, London, Southampton, Manchester, Taunton, Gloucester, Sheffield, Bath, and Exeter—via the local child health records offices that organise routine childhood vaccination appointments. Household family units provided full consent and were enrolled into the study early in the LAIV immunisation period (September–November), and the study was carried out from Oct 3, 2017 to Jan 4, 2018 and Sept 27, 2018 to Jan 2, 2019.

Index children were given a quadrivalent LAIV with two A and two B strains. Two strains were modified between the two seasons. During the first season, the strains comprising the vaccine were^9^ an A/Michigan/45/2015 (H1N1)pdm09-like virus, an A/Hong Kong/4801/2014 (H3N2)-like virus, a B/Brisbane/60/2008-like virus, and a B/Phuket/3073/2013-like virus (Yamagata-lineage). During the second season, the strains were an A/Michigan/45/2015 (H1N1)pdm09-like strain, an A/Singapore/INFIMH-16-0019/2016 (H3N2)-like strain, a B/Colorado/60/2017-like strain, and a B/Phuket/3073/2013-like strain.^10^

Owing to an administrative mix-up and cancellation of the initial prospective International Standard Randomised Controlled Trial Number (ISRCTN) application, ISRCTN registration was only completed in 2019, 2 years after the study had started. Despite retrospective registration, the trial was conducted according to the protocol, as finalised in 2017 and as approved by the ethics committee before study commencement. Some Integrated Research Application System approved amendments were made between the sampling periods, and these are detailed in the Health Research Authority (HRA) approval letter (appendix p 37).

Ethical and governance approvals were granted by the UK Research Ethics Committee (Cambridgeshire and Hertfordshire; 17/EE/0351), the HRA (17/SW/0190), and the local NHS Research and Development departments at each site. All participants or their parents or carers provided written informed consent either in writing or by an electronic process. For children younger than 16 years, parental consent was taken, and when appropriate, assent was sought.

This study followed the CONSORT guidelines.

### Randomisation and masking

After informed consent was obtained, LD allocated each family a study number, which was used for randomisation with equal allocation (1:1) between either the early LAIV group, in which the 2-year-old index child was immunised with LAIV at visit 1 (after baseline sample collection), or to the late LAIV group (control), in which the index child did not receive LAIV until 28 days later, at visit 3 (after visit 3 sample collection). The allocation was done by JO and the study nurses using a single central allocation service with computerised randomisation for all sites. Allocation concealment and masking was not possible in this trial.

### Procedures

All participating households had five study visits (visits 1 to 5) either at home or in a clinic, at 14-day intervals, to collect nasopharyngeal samples from each participant at each visit (appendix p 4). Demographic data as reported by parents (ie, age, sex, household size, daycare or school attendance, and vaccination status of the index child) were collected at baseline; clinical data on current or recent (within 7 days) antibiotic use and self-assessed coryzal status according to the Symptoms of Nasal Outflow Tally (scored 0–3), as previously defined,^11^ were collected at each study visit. At enrolment (day 0, visit 1), household family units were randomly allocated (1:1) to either the early or late LAIV group. Household members eligible for LAIV, other than the index child, were asked to defer LAIV until all five visits and nasopharyngeal samples had been collected.

Nasopharyngeal samples were taken with PurFlock Ultra swabs (Medical Wire & Equipment, Corsham, UK) using a standardised method (appendix pp 16–36), transported in 1·5 mL STGG medium (prepared in-house; Germantown, MD, USA) at 2–8°C, and frozen within 4 h. All samples were analysed for the presence of *S pneumoniae* by quantitative PCR with *lytA* as the gene target, as previously described, with a cycle threshold (Ct) cutoff of 35 and Ct values converted to gene copies per mL (gc/mL) using a standard curve.^11^ In this study, *lytA* detection at a Ct less than 35 is referred to as *S pneumoniae* positivity. Further details of sample collection, nucleic acid extraction, quantitative PCR set-up, and analysis, including primer and probe sequences, control strategies, and instrument settings, are provided in the appendix (pp 12–15).

### Outcomes

The change in nasopharyngeal density of *S pneumoniae* in index children, as established by quantitative PCR at 14 and 28 days after LAIV, compared with that on day 0, was defined as a coprimary outcome in the clinical trial protocol. Another coprimary outcome was the prevalence of *S pneumoniae* colonisation at days 14 and 28 in household members who were *S pneumoniae*-negative at day 0 but whose index children were *S pneumoniae-*positive at day 0.

In this Article, we report the effects of LAIV on the biology of *S pneumoniae* colonisation and acquisition of *S pneumoniae* in household contacts. Secondary outcomes of the trial, which include the colonisation in index children and acquisition in household contacts of two other pathogens (ie, *Moraxella catarrhalis* and *Haemophilus influenzae*), will be published elsewhere.

### Safety monitoring

LAIV was used within its licensed indication, dose and route, and was administered only to children who were eligible to receive LAIV through the routine NHS childhood influenza immunisation programme. The trial was designed to assess the effect of LAIV on nasopharyngeal pneumococcal carriage; vaccine safety was not a primary or secondary outcome.

Safety monitoring was therefore proportionate to the established safety profile of LAIV and the low-risk nature of the additional research procedures. Expected, non-serious post-vaccination symptoms were not actively solicited or systematically collected as trial outcomes. Serious adverse events were recorded if reported to the study team and were assessed by the Chief Investigator for seriousness, expectedness, and relatedness to LAIV or study procedures. Events considered related and unexpected were to be reported in accordance with sponsor and regulatory requirements.

### Statistical analysis

There was no independent data and safety monitoring board because the intervention was a licensed vaccine being given according to the routine schedule for children of this age at the time.

At the time of the study design, the probable effect size of LAIV on *S pneumoniae* transmission events was unknown. Assumptions and predictions made to calculate sample size were as follows: at least 50% of the index participants would be *S pneumoniae* carriers, rendering half of the overall sample of families informative for the primary objective. At baseline, more than 15% of household contacts (the probable principal contacts of the children) were expected to be carriers of *S pneumoniae*, with a carriage rate of at least 9% in parents and up to 60% in other children in the household.^12^ From the UK census data,^13^ a child aged 2 years had a mean of 2·46 household contacts. On the basis of these assumptions, we calculated the sample size required to detect a two-times increase in presumed transmission with 90% power, allowing for a 20% dropout rate.

Detecting a two-times rise in *S pneumoniae* acquisition with 90% power necessitated a sample size of 260 household contacts of children carrying *S pneumoniae* at the outset per study arm, thus totalling 520 contacts of carriers. Assuming the average 2·46 household contacts, the study required recruitment of 212 households. If half of the approached households were non-informative (ie., children to be vaccinated were not carriers of *S pneumoniae* at the outset), then 424 households were needed to achieve the required 520 contacts for the primary objective. Accordingly, a study size of 500 children and their families, yielding 1230 contacts, was deemed sufficient to detect the effect for the primary objective, allowing for a 20% dropout rate. The final number of families recruited during the two seasons was 405, 95 fewer than planned. A third season was considered, but the much higher study-completion rate of participating families (97% rather than 80% as predicted) and preliminary data indicating a substantially higher rate of baseline *S pneumoniae* carriage in index participants (68% rather than 50%, as predicted for the power calculations) reassured us that the recruited 405 families would be sufficient to answer the study objectives.

Baseline comparisons between participant groups were carried out using all the samples taken. An intention-to-treat analysis using all available data from participants with valid consent, irrespective of whether participants provided a sample at every visit, was used for *S pneumoniae* point prevalence. Results from children and their contacts with complete data (results available from every visit) were used for the per-protocol analysis; index children positive for *S pneumoniae* at baseline, together with their discordant (negative for *S pneumoniae* at baseline) contacts, were included for the analysis of *S pneumoniae* acquisition. The relative risk of the contact being positive for *S pneumoniae* when the index child was positive was calculated. Prevalence proportions are reported as percentages with associated binomial CIs. The Mann–Whitney test was used to assess whether the change in *S pneumoniae* density between visits 1 and 2 in index children who were in the early LAIV group differed significantly from changes in density in index children in the control late LAIV group.

We used two complementary approaches to analyse the data. First, we used statistical tests. Chi squared test was used to compare *S pneumoniae* carriage point prevalence and changes in presumed transmission data, and Mann–Whitney U test was used to compare densities. Second, to ensure robustness of results and take into account the wide natural variability in carriage density,^7^ we also performed regression analyses: we developed a simple logistic regression model to analyse the association between vaccination with LAIV (binary explanatory variable) and whether an increase in pneumococcal density 14 and 28 days later was observed (outcome variable with two categories: an observed increase or non-increase in *S pneumoniae* density at visit 2). The Chi squared test was used to assess whether a change occurred in the odds of transmission to household members after administering LAIV, which was further confirmed using a logistic regression model. As a post-hoc analysis, a mixed-effects logistic regression model of *S pneumoniae* acquisition in contacts, with random intercepts to account for correlations among contacts of index children from the same household, was developed as a sensitivity analysis. The fixed-effects and mixed-effects models were compared using the Akaike Information Criterion (AIC). For all logistic regression models, we estimated the odds ratio and the 95% CIs, with a p value less than 0·05 being the threshold for significance.

Finally, we performed a further post-hoc analysis to consider the possibility of a density-independent effect of LAIV on *S pneumoniae* acquisition in contacts. To evaluate this possibility, we assessed the median carriage density of baseline-positive index participants at visit 2 whose contacts had become positive at visit 2 and compared these between the two study groups.

All analyses were carried out in R version 4.2.3.

### Role of the funding source

The funder of the study had no other role in study design, data collection, data analysis, or writing of the report.

## Results

We enrolled 405 families (1379 participants); 205 families (and index children) were randomly assigned to the early LAIV group, and 200 families (and index children) were assigned to the late LAIV group. Three families withdrew before completion of visit 1 and were not included in subsequent study analysis. 388 (96%) of 405 enrolled households, including 1309 (95%) of 1379 enrolled participants completed the study (figure 1). The study was conducted during two consecutive LAIV-vaccinating seasons; 1985 samples from 422 participants were collected between Oct 3, 2017, and Jan 4, 2018 (season 1) and 4495 samples from 957 participants between Sept 27, 2018, and Jan 2, 2019 (season 2). Baseline characteristics of index children are shown in table 1. In the early LAIV group, 3 of 205 participants had missing demographic data; 116 (57%) of the remaining 202 index children were female and 86 (43%) were male, and the median age was 2·47 years (IQR 2·32–2·65); in the late LAIV group, 91 (46%) of 200 index children were female and 108 (54%) were male, and the median age was 2·50 years (IQR 2·30–2·64). In the early LAIV group, 276 (59%) of 470 household contacts were female and 194 (41%) were male and the median age of contacts was 33·8 (IQR 31·7); in the late LAIV group 272 (60%) of 453 household contacts were female and 181 (40%) were male and the median age of contacts was 33·1 (IQR 31·4). There were no significant differences in demographics between the household contacts in the two study groups (table 2).

**Figure 1:**
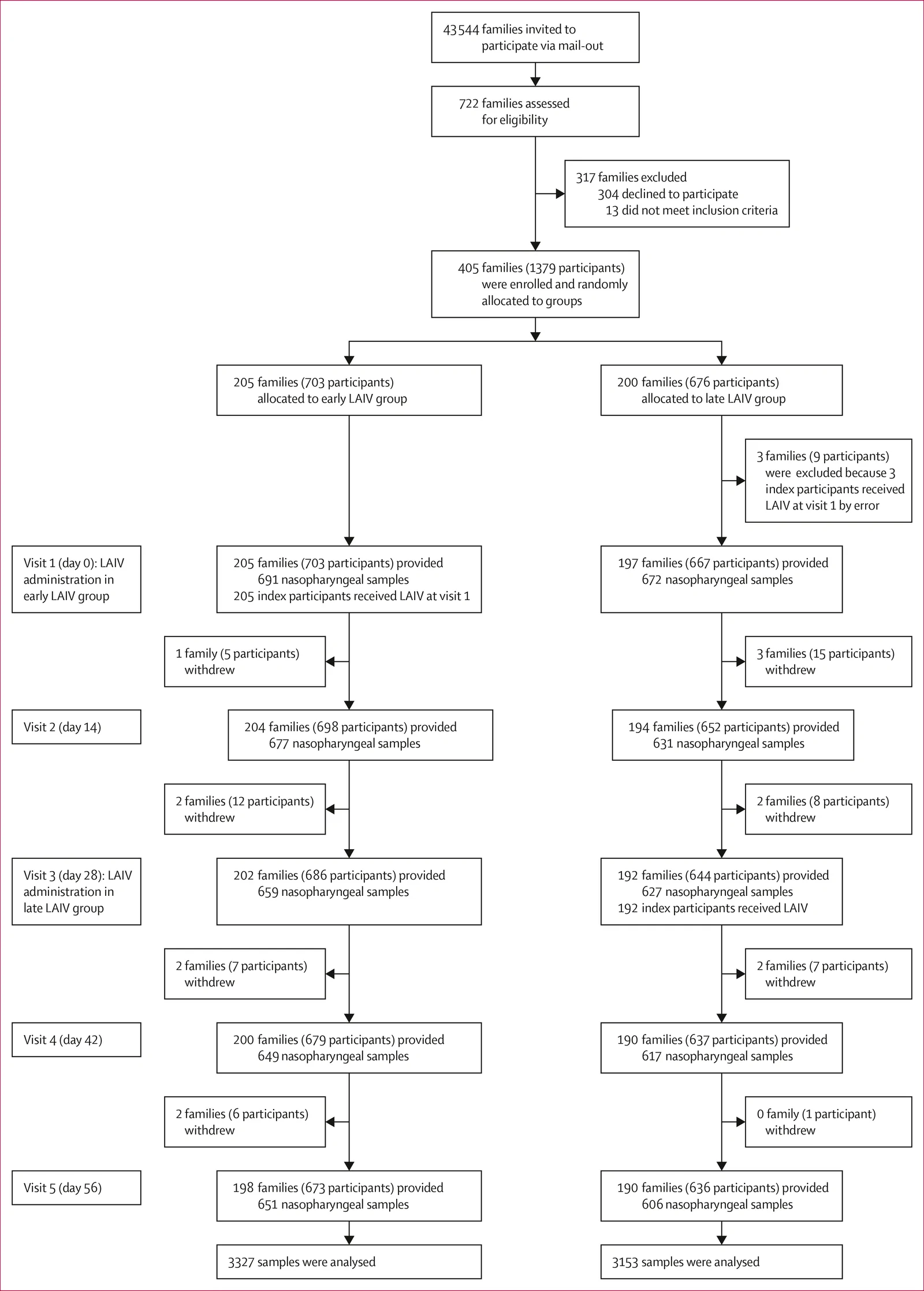
Trial profile LAIV=live attenuated influenza vaccine.

**Table 1:** Demographic and clinical characteristics of the index participants at baseline

	Early LAIV group (n=202)∗	Control late LAIV group (n=200)
Age, years	2·47 (2·32–2·65)	2·50 (2·30–2·64)
Sex		
Male	86 (43%)	108 (54%)
Female	116 (57%)	91 (46%)
Daycare attendance	150 (74%)	150 (75%)
Household size	3·94 (0·78)	3·92 (0·77)
SNOT score†	0·62 (0·70)	0·67 (0·78)
SNOT score >0)	102 (51%)	99 (50%)
Antibiotic score‡	1·02 (0·14)	1·04 (0·24)

Data are median (IQR), n (%), or mean (SD). Data on race/ethnicity were not collected during the study. No formal hypothesis testing was done, as families (so index children) were the unit of randomisation. LAIV=live attenuated influenza vaccine. SNOT=Symptoms of Nasal Outflow Tally.

∗ Three index participants are not included in this table, as they were missing demographic or clinical information.

† SNOT scores: 0=no coryzal symptoms; 1=mild; 2=moderate; and 3=severe.

‡ Antibiotic score: 1=no antibiotics taken in the past 7 days or currently; 2=antibiotics taken in the past 7 days; and 3=currently taking antibiotics.

**Table 2:** Comparison of demographic or clinical characteristics between contacts of index children at visit 1

	Children aged 5 years and younger∗			Children aged 5–17 years			Adults (18 years and older)		
	Early LAIV group (n=73)	Late LAIV group (n=69)	p value	Early LAIV group (n=76)	Late LAIV group (n=74)	p value	Early LAIV group (n=322)	Late LAIV group (n=309)	p value
Age, years∼	2·6 (0·7–4·4)	3·8 (0·8–4·5)	0·40	6·7 (5·5–9·1)	6·5 (5·5–8·5)	0·40	37·4 (33·6–40·4)	36·5 (32·8–39·6)	0·06
Sex									
Female	32 (44%)	38† (56%)	⋅⋅	39‡ (52%)	39§ (53%)	⋅⋅	205 (64%)	195¶ (63%)	⋅⋅
Male	41 (56%)	30† (44%)	0·20‖	36‡ (48%)	34§ (47%)	0·90‖	117 (36%)	113¶ (37%)	0·90‖
Daycare attendance	13 (18%)	13 (19%)	0·90‖	0	0	··	1 (0·3%)	4 (1·3%)	0·20‖
Household size	4·30 (0·74)	4·22 (0·66)	0·60	4·53 (0·81)	4·46 (0·80)	0·80	3·91 (0·77)	3·90 (0·88)	0·90
SNOT score‖	0·53 (0·67)	0·66 (0·79)	0·40	0·40 (0·59)	0·36 (0·64)	0·60	0·26 (0·50)	0·35 (0·63)	0·20
SNOT score >0	31 (43%)	32 (46%)	0·60‖	26 (34%)	21 (28%)	0·50‖	75 (23%)	85 (28%)	0·20‖
Antibiotic score∗∗	1·07 (0·35)	1·03 (0·17)	0·70	1·03 (0·16)	1·00 (0·00)	0·20	1·03 (0·25)	1·04 (0·23)	0·20

Data are median (IQR), n (%), or mean (SD). Statistical analysis testing was done using the Chi squared test for categorical data (sex, daycare attendance, and SNOT score >0), independent *t* test for normally distributed continuous data (∼), and Mann–Whitney test for non-normally distributed continuous data (household size, SNOT score, and antibiotic score). LAIV=live attenuated influenza vaccine. SNOT=Symptoms of Nasal Outflow Tally.

∗ Three index participants are not included in this table as they were missing demographic or clinical information.

† Sex data not available for 1/69 of the children aged 5 years and younger in the Late LAIV group

‡ Sex data not available for 1/76 of the children aged 5–17 years in the Early LAIV group.

§ Sex data not available for 1/74 Late LAIV group.

¶ Sex data not available for 1/309 of the Adults in the Late LAIV group.

‖ SNOT scores: 0=no coryzal symptoms; 1=mild; 2=moderate; and 3=severe.

∗∗ Antibiotic score: 1=no antibiotics taken in the past 7 days or currently; 2=antibiotics taken in the past 7 days; and 3=currently taking antibiotics.

1363 (98·8%) of 1379 enrolled participants provided a baseline (visit 1) sample, including 550 children younger than 5 years (of whom 405 were index participants), 159 children aged 5–17 years, and 654 adults (≥18 years; appendix p 5). Visit 1 occurred between Oct 3, 2017, and Oct 31, 2017 (123 families enrolled), or Sept 27, 2018, and Nov 2, 2018 (282 families enrolled). Young children under the age of 5 years carried *S pneumoniae* more frequently and at higher densities than children over 5 years and adults household members (figure 2). 276 (68%) of 405 index children and 227 (24%) of 958 contacts were carriers at baseline. Antibiotic use (current or within the past week) was minimal across all participants (table 1). *S pneumoniae* density followed a skewed distribution in index children, with variation over six orders of magnitude (3·5 gc/mL to 1·3 × 10^6^ gc/mL; median 1911 gc/mL [IQR 288–10 424]; figure 2). Additionally, *S pneumoniae* density in index children was similar between the two groups at the first visit, with a median density of 4744 gc/mL in the late LAIV group and 5657 gc/mL in the early LAIV group (Mann–Whitney U test p=0·64).

**Figure 2:**
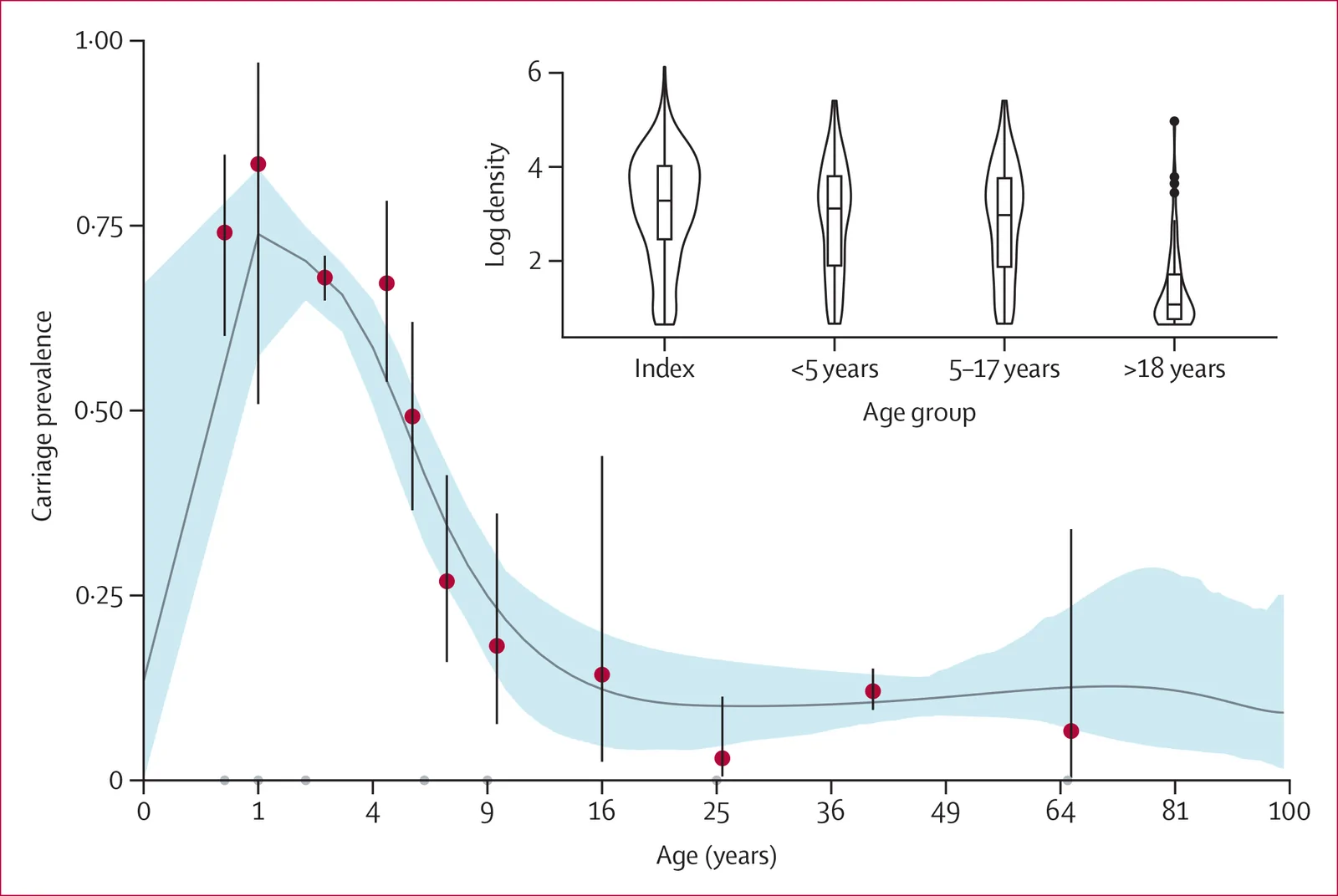
Pneumococcal carriage and density (inset) as a function of age Red dots represent the observed carriage point prevalence, black vertical bars the 95% binomial CIs, black curve the spline fit, and blue shaded area the 95% credible intervals of the spline. Grey dots indicate positions of the knots. Inset: Violin plots show the log carriage density among index children and their contacts at visit 1. The boxplots nested within show the quartiles of these log densities. Methodology for the spline fit and Bayesian fitting can be found elsewhere.^14^

On comparing study groups at baseline (before any intervention), 139 of 205 (68%, 95% CI 61–74) index children in the early LAIV group and 136 of 200 (68%, 61–74) index children in the control late LAIV group were *S pneumoniae* carriers. 100 of 486 (21%, 95% CI 19–27) household contacts in the early LAIV group and 118 of 472 (25%, 21–29) household contacts in the control late LAIV group were *S pneumoniae* carriers.

At baseline, contacts of index children who were *S pneumoniae*-positive (181 [28%] of 652) were significantly more likely to also be *S pneumoniae* carriers than were contacts of index children who were *S pneumoniae*-negative (46 [15%] of 306; p value=0·0001). The relative risk of the contact being positive for *S pneumoniae* when the index child was positive was 1·85 (95% CI 1·38–2·49).

245 (89%) of the 275 index children who were positive for *S pneumoniae* at baseline had complete data available for all five visits and were included in the analysis to investigate the effect of LAIV on carriage density. An increase in density was observed in 62 of 128 (48%, 95% CI 40–57) of the index children who were baseline-positive and had received LAIV at visit 1, 14 days after immunisation, and in 32 of 117 (27%, 20–36) of those who had not received LAIV (Chi squared test p=0·0007; appendix pp 7–8). This finding was further confirmed by the logistic regression model (table 3). The magnitude of these density increases was larger in the group that had received LAIV (median change in density increase 6020 gc/mL) than in the group that had not (3440 gc/mL), although the difference was not significant (Mann–Whitney U test p=0·25; appendix p 8). A stronger effect was observed when considering only index participants who were *S pneumoniae* carriers at both visits 1 and 2: the median change in density between visit 1 and 14 days later at visit 2 was +854 gc/mL in LAIV recipients (early LAIV group) and –393 gc/mL in LAIV non-recipients (control late LAIV group; Mann–Whitney U test p=0·017). By visit 3, 28 days following LAIV, there was little evidence of a persistent increase in *S pneumoniae* density relative to baseline (visit 1) among LAIV recipients compared with non-recipients (table 3). These data indicate that LAIV achieved the intended outcome of transiently increasing *S pneumoniae* density, allowing us to further assess, in the context of this increase, the effect of LAIV in acquisition in household contacts.

**Table 3:** Univariate logistic regression analyses

	Odds ratio (95% CI)	p value
**Increase in density in index children**		
From visit 1 to visit 2	2·5 (1·3–4·3)	<0·0008
From visit 1 to visit 3	1·5 (0·88–2·5)	0·14
***S pneumoniae* acquisition in household contacts**		
Discordant pairs at visit 2	2·0 (1·2–3·5)	0·015
Discordant pairs at visit 3	0·89 (0·53–1·5)	0·67

Univariate logistic regression analyses of increase in carriage density between index children in the early and late LAIV groups, from visit 1 to visit 2 and from visit 1 to visit 3; and *Streptococcus pneumoniae* acquisition events in household contacts of index children who were *S pneumoniae*-positive at baseline in the early vs late LAIV groups at visit 2 (day 14) and visit 3 (day 28). At visit 3, we considered family contacts only when they had been *S pneumoniae*-negative at both visits 1 and 2.

LAIV=live attenuated influenza vaccine.

Irrespective of the index child’s baseline carriage status, 59 of 367 (16%, 95% CI 13–20) contacts in the early LAIV group who were pneumococcal carriage-negative at baseline became positive by 14 days after LAIV vaccination of the index child. In the late LAIV group, 31 of 329 (9%, 95% CI 7–13) contacts became positive within the same period (Chi squared test p=0·0090). By the time of the third study visit at 28 days (when *S pneumoniae* carriage density was similar among index children that had and had not received LAIV), there was no discernible difference in *S pneumoniae* acquisition rates between contacts in the early LAIV group (57 of 359; 16%, 95% CI 12–20) and contacts in the late LAIV group (49 of 313; 16%, 12–20; p=0·94).

When we restricted the analysis to index children who were *S pneumoniae* carriage-positive at baseline and their contacts who were *S pneumoniae* carriage-negative at baseline, we found 445 discordant contacts from 251 index children. 67 (15%) of these 445 contacts were *S pneumoniae*-positive at visit 2 (14 days later), including 45 (19%) of 237 in the early LAIV group and 22 (11%) of 208 in the late LAIV group (p=0·013). Of the 45 contacts in the early LAIV group who became *S pneumoniae*-positive, eight were aged 5 years or younger, four were aged 6–17 years, and 33 were aged 18 years or older. Of the 22 contacts in the late LAIV group who became *S pneumoniae*-positive, five were aged 5 years or younger, one was aged 6–17 years, and 16 were aged 18 years or older. Acquisition events between discordant pairs and their contacts were restricted to one event in 62 (97%) of the 64 households wherein these events occurred. Chi squared analysis and both fixed-effects and mixed-effects regression analyses of *S pneumoniae* acquisition in these families showed that the odds of detecting *S pneumoniae* acquisition in household members were two times higher at visit 2 in families in the early LAIV group than in control families (table 3). Augmenting the simple logistic regression model to include random effects among contacts within the same family showed an odds ratio similar to that seen with the fixed-effects model (appendix p 3); the AIC of the simple logistic regression model was 377, whereas that of the fixed-effects model was 375.

By visit 3, on day 28, no significant difference in the point prevalence of *S pneumoniae* carriage was observed between contacts in the early LAIV group (35 of 226; 15%, 95% CI 11–21) and contacts in the late LAIV group (34 of 200; 17%, 12–22; p=0·67). Both fixed-effects and mixed-effects logistic regression showed that there were no significant differences in the rates of acquisition events detected at visit 3 (28 days) between the two study groups among families whose index child carried pneumococcus at baseline but whose contacts were negative at both visits 1 and 2 (table 3; appendix p 3). We also found that of the 67 contacts among the pairs discordant at baseline who were *S pneumoniae*-positive at visit 2 (45 from the early LAIV group and 22 from the late LAIV group), 23 (34%) remained positive at visit 3 (14 [31%] of 45 from the early LAIV group and 9 [41%] of 22 from the late LAIV group); again, no significant difference was observed between these contacts in the two groups (Chi squared test p=0·43).

As additional support for the data from the randomised control trial, in post-hoc analysis we assessed across both the study arms whether *S pneumoniae* carriage density predicted household acquisition, using an observational retrospective analysis of visit-2 samples. The carriage density in index children one or more contacts of whom had acquired *S pneumoniae* (median 5187 gc/mL [IQR: 12 469 gc/mL]) was significantly higher than in index children whose contacts all remained *S pneumoniae* carriage-negative (1536 gc/mL [IQR: 8196 gc/mL]; Mann–Whitney U test p=0·029). Upon separating study groups, a difference in density remained in the early LAIV group: in this group, the median density in index children at least one contact of whom had acquired *S pneumoniae* was 4845 gc/mL (A: IQR: 15 994 gc/mL), compared with 981 gc/mL (A: IQR: 8100 gc/mL) in those whose contacts remained *S pneumoniae* carriage-negative (Mann–Whitney U test p=0·026). In the late LAIV group, the median density was 5592 gc/mL [A: IQR: 6497 gc/mL] in index children at least one contact of whom had acquired *S pneumoniae*, as compared with 2008 gc/mL (A: IQR: 10 646 gc/mL) in index participants whose contacts remained *S pneumoniae*-negative (Mann–Whitney U test p=0·28; appendix p 9).

Three serious adverse events were reported during the study. These events occurred in the context of pregnancy or breastfeeding and were assessed as unrelated to LAIV or to study procedures. No serious adverse events were considered related to LAIV administration, nasopharyngeal swabbing, or other study procedures. No suspected unexpected serious adverse reactions were reported.

## Discussion

Intranasal administration of attenuated influenza virus strains transiently increased detectable bacterial load in individuals colonised with *S pneumoniae,* as shown previously in studies by our group and others,.^6^^,^^8^^,^^15^ In this study, we took samples at 14-day intervals and found this rise to occur at 14 days but to have largely dissipated by 28 days.

We found that in the context of a modest average rise in nasal *S pneumoniae* density, the odds of *S pneumoniae* acquisition in household contacts who were baseline *S pneumoniae*-negative were temporarily higher in the study group in which index children received LAIV than in the group in which children did not receive LAIV. This finding is notwithstanding the great background diversity of *S pneumoniae* carriage density that existed among these young children. Further analysis of samples to identify pneumococcal serotype and exploration of contact patterns within and outside the families would help to confirm the extent to which these differences could have been diluted by other transmission events. However, as this study was a randomised controlled trial, concluding that the observed excess acquisition in contacts of LAIV recipients was causally related is reasonable.

Taken together, our results suggest that an attenuated influenza virus infection increases the likelihood of transmission of pneumococcus from young children who are colonised with the bacterium to their home contacts and that this increase is driven largely by an increase in bacterial abundance triggered by the influenza vaccine. Although there could be other mechanisms by which administration of attenuated influenza viruses to children aged 2 years might lead to increases in nasal *lytA* positivity in their family contacts, a transient increase in infectiousness of the vaccine recipients who were colonised with *S pneumoniae* appears to be the most likely explanation. This observation raises several new questions about the nature of the dynamics of the bacterial density, the transmission relationship, and the mechanisms involved, particularly how the bacteria sense the viral infection and whether the host response to the vaccine is involved. These aspects will require further investigation.

The implications of these findings for pneumococcal ecology at the population level cannot be deduced from the results of this small study. On one hand, the observed vaccine-induced increases in colonisation density and rates of *S pneumoniae* acquisition in the contacts were modest and transient as compared with the wide range of densities we observed and increases in bacterial densities that have been described in association with common wild-type respiratory viral infections.^16^ On the other hand, in the UK, LAIV is administered to large numbers of children over a quite short period of time within the last quarter of each year and the first quarter of the following year. Given the wellaccepted importance of children in the dynamics of pneumococcal transmission across all age groups, this question deserves further attention.

The main strength of this study lies in its randomised controlled design, reducing potential biases and permitting causality to be inferred with regard to the findings. A limitation was detection of *S pneumoniae* only by its autolysin gene rather than also by serotype confirmation, reducing the confidence in the inference that an acquisition event from study child to family member occurred for any individual child. At the analytical level of the full study population, the randomised design should have protected against bias consequent upon any such errors. The two-week gap between sampling reduced the precision with which the density changes and acquisition events could be tracked, and ideally, more frequent testing would have occurred; however, we were constrained to a frequency that participating families would accept. Another potential limitation is the possibility that the *lytA* target can also detect some strains of non-pneumococcal alpha streptococci, which can also occasionally be found in nasal samples. Accordingly, our findings, although predominantly reflecting pneumococcal carriage and transmission, could also include other closely related bacterial species to a small degree. A further limitation is that we did not report on the prevalence of intercurrent respiratory viral infections in the index children and their family contacts during the study, which would undoubtedly have contributed to the observed wide diversity of bacterial colonisation density and acquisition in both the groups.^16^ However, as this is a randomised control trial, we assume that viral acquisition is similar between the two groups.

As acquisition events between discordant pairs were mostly restricted to one event per household, a fixed-effects model was found to be sufficient, with a lower AIC than for the mixed-effects model. However, the number of transmission events at visit 3 was much more widely distributed than at visit 2. Hence, the mixed-effects model outperformed its fixed-effects counterpart in terms of the respective AIC values.

Aside from providing new insights into the complex interactions among viruses, bacteria, and the human host in the upper respiratory tract, the findings of this study add credence to the hypothesis that the control of symptomatic and invasive infections caused by colonising pathobiont bacteria could be achieved to some degree through prevention of respiratory viral infections with mucosal vaccines, assuming vaccines can provide sterilising immunity or, more likely, reduction of viral load during infection. Our results, in turn, are consistent with observations about the relationships between respiratory viral infections and bacterial infections made possible by the large disruptions in their epidemiology brought about by measures taken to reduce transmission of SARS-CoV-2 during the COVID-19 pandemic, including closure of schools.^17^ Taken together, these data emphasise the need to improve understanding of viral–bacterial interactions in the respiratory tract, to improve upon measures to prevent disease and optimise immunity.

In summary, our study confirms that LAIV significantly increases *S pneumoniae* carriage density in previously LAIV-naive recipient young children and transiently increases presumed household *S pneumoniae* transmission. Despite the modest density increase, household *S pneumoniae* transmission events increased, emphasising the potential for respiratory viruses to transiently drive population-level *S pneumoniae* carriage, probably through triggering *S pneumoniae* replication. Whole-data mixed-group analysis comparing vaccine exposed and unexposed periods produced more borderline results, suggesting that other covariates could also affect *S pneumoniae* transmission. These results highlight important knowledge gaps in the understanding of *S pneumoniae* transmission, which warrant further investigation and suggest that respiratory viral infections increase upper airway bacterial transmission; and hence, preventing them through vaccination might play a beneficial role in reducing diseases caused by these bacteria.

## Data sharing

Study data cannot be made publicly available but may be provided upon formal request by e-mailing Leon Danon at l.danon@bristol.ac.uk.

## Declaration of interests

LD is funded by the UK Research and Innovation AI programme of the Engineering and Physical Sciences Research Council (EP/Y028392/1) and is affiliated with the JUNIPER (Joint UNIversities Pandemic and Epidemiological Research) partnership funded by the Medical Research Council (MR/X018598/1). LD also receives research funding from Pfizer via the AvonCAP study and works on a separate study funded by Pfizer. AF undertakes paid consultancy for several influenza vaccine companies, including AstraZeneca (who market a live attenuated influenza vaccine), GSK, Sanofi, and Seqirus. AF is Chief Investigator on this study and another project, both funded by Pfizer. BG was an employee of Pfizer when the work was conducted and holds Pfizer stock. SF, as an institutional investigator (with no personal payment), received grants or contracts with Pfizer, Sanofi, GSK, Johnson & Johnson, Merck, AstraZeneca, Valneva, Moderna, and BioNTech, but no royalties, licences, or consulting fees. SF’s institution received symposium fees from Moderna, Novavax, and Pfizer (none to SF personally). SF served on data and safety monitoring boards or advisory boards for AstraZeneca, MedImmune, Sanofi, Pfizer, Seqirus, Merck, Johnson & Johnson, and MSD (fees to institution). During the study, MS received institutional funding and no personal payment for vaccine trials from Janssen, GSK, AstraZeneca, and Pfizer. Since 2022, MS is an employee of, and holds equity within, Moderna. All other authors declare no competing interests.
