# Supplementary material for: Effects of attenuated influenza virus administration to young children on pneumococcal density and acquisition in UK families: a randomised controlled trial
Source: Lancet Microbe. 2026 Oct;7(10):None. doi: 10.1016/j.lanmic.2026.101422 (PMC13633236; doi:10.1016/j.lanmic.2026.101422)
Supplement: Supplementary file 1 [file mmc1.pdf]

THE LANCET
Microbe

Supplementary appendix

This appendix formed part of the original submission and has been peer reviewed.
We post it as supplied by the authors.

Supplement to: Metz J, Qian G, Morales-Aza B, et al. Effects of attenuated influenza virus administration to young children on pneumococcal density and acquisition in UK families: a randomised controlled trial. *Lancet Microbe* 2026. https://doi.org/10.1016/j.lanmic.2026.101422

Appendix to: *The effect of attenuated influenza virus administration to young children on pneumococcal density and transmission in UK families: a randomised controlled trial*

Table of Contents

Table A1: Recruitment, enrolment, randomisation, withdrawal and completion of study by participating families and participants.......................................**2**

Table A2: Logistic regression mixed-effects model comparing Sp acquisition events from 2-year-old index children carrying pneumococcus at baseline to family contacts at visit 2 (14 days) and visit 3 (28 days) between the early and late LAIV group..............................**3**

Figure A1: Study timeline for data and sample collection and LAIV administration .. **4**

Figure A2: Age and frequency of the contacts of 2-year-old index children from the early and late (control) LAIV study arms...**5**

Figure A3: The dynamics of pneumococcal carriage density in visit 1-positive index children across all 5 visits..**6**

Figure A4: The dynamics of pneumococcal carriage density in visit 1-negative index children across all 5 visits..**7**

Figure A5: Change in pneumococcal carriage density by arm from visit 1 to visit 2.....**8**

Figure A6: Carriage density of index children at visit 2 (14 days after LAIV in Early LAIV group) plotted according to whether an acquisition event had occurred................. **9**

Inclusion/Exclusion Criteria...**10**

Sample collection, nucleic acid extraction, qPCR set-up and analysis....12

Study protocol...16

Amendment letter...37

Table A1: Recruitment, enrolment, randomisation, withdrawal and completion of study by participating families and participants according to season and study site.

Site	Recruitment		Enrolment			Randomisation		Withdrawal		Completion (main study)		
	Mail-out	Screened	Families	Participants	Family size	Group 1 families (participants)	Group 2 families (participants)	Families (gp1:gp2)	Participants	Families (%)	Participants	Contacts
**Bristol S1**	6000	100	50	184	3.68	25 (92)	25(92)	1 (0:1)	7	49 (98%)	177	128
**St Georges S1**	3000	38	25	80	3.2	12 (39)	13 (41)	0	0	25 (100%)	80	55
**Manchester S1**	5000	53	17	55	3.24	12 (38)	5 (17)	0	0	17 (100%)	55	38
**Oxford S1**	19	13	9	28	3.11	5 (15)	4 (13)	0	1	9 (100%)	27	18
**Southampton S1**	2300	50	22	75	3.41	9 (33)	13 (42)	2 (1:1)	7	20 (91%)	68	48
**Subtotal season 1**	16319	254	123	422	3.43	63 (217)	60 (205)	3	15	120 (97%)	407	287
**Bristol S2**	3155	100	53	195	3.68	31 (117)	22 (78)	3 (2:1):	16 (11:5)	50 (94%)	179	129
**St Georges S2**	6500	67	42	128	3.05	21 (63)	21 (65)	0	0	42 (100%)	128	86
**Southampton S2**	1800	31	22	70	3.18	13 (41)	9 (29)	2 (1:1)	6 (3:3)	20 (91%)	64	44
**Manchester S2**	5000	50	20	69	3.45	9 (30)	11 (39)	1 (0:1)	3 (0:3)	19 (95%)	66	47
**Oxford S2**	4200	65	42	132	3.14	19 (59)	23 (73)	1 (1:0)	4 (4:0)	41 (95%)	128	87
**Sheffield S2**	2000	32	19	65	3.42	7 (24)	12 (41)	0	0	19 (95%)	65	46
**Gloucester S2**	1037	31	19	70	3.68	11 (39)	8 (31)	0	0	19 (100%)	70	51
**Exeter S2**	1529	27	20	69	3.45	9 (34)	11 (35)	1 (1:0)	3 (3:0)	19 (95%)	66	47
**Bath S2**	855	36	21	81	3.86	11 (43)	10 (38)	3 (1:2)	14 (5:9)	18 (86%)	67	49
**Taunton S2**	1149	29	24	78	3.25	11 (36)	13 (42)	0	0	24 (100%)	78	54
**Subtotal season 2**	27225	468	282	957	3.39	142 (486)	140 (471)	11 (6:5)	46	271 (96%)	911	640
**Total**	43544	722	405	1379	3.41	205	200	14	61	391 (97%)	1318	927

Table A2: Logistic regression mixed-effects model comparing Sp acquisition events from 2-year-old index children carrying pneumococcus at baseline to family contacts at visit 2 (14 days) and visit 3 (28 days) between the early and late LAIV groups. We used a random intercept model to account for random effects between households participating in the study. In the early group, children received LAIV at visit 1 after baseline sampling, while in the late group, children did not receive LAIV until after sampling at visit 3.

Visits assessed	Odds Ratio	95% CIs	p-value	S.D. of random effects	AIC of fixed effects model	AIC of mixed effects model
From visit 1 to visit 2	2.0	1.0 – 3.8	0.036	0.26	375	377
From visit 1 to visit 3	0.85	0.45 – 1.6	0.61	1.1	381	380

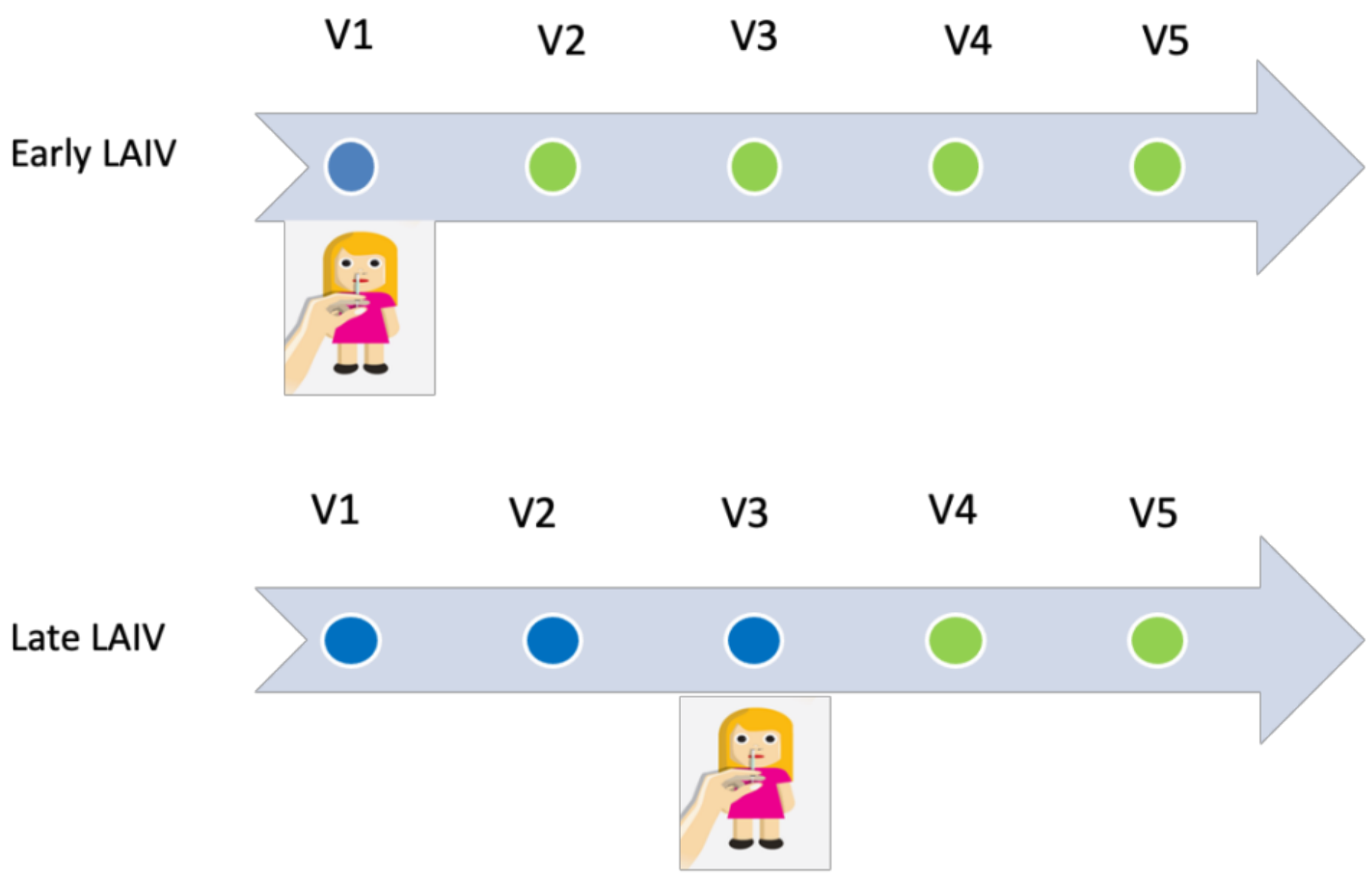

Figure A1. Study timeline for data and sample collection and LAIV administration. Baseline demographic data was collected at Visit 1 (V1). Clinical data and NP samples were collected from all participants at each study visit (V1-V5). In household families randomised into the early LAIV group, the index child received LAIV after samples were taken at visit 1. In household families randomised into the late LAIV group, the index child received LAIV after samples were taken at visit 3. The blue and green spots indicate study sampling times, blue, where index participants were LAIV-naïve and green where LAIV administration to index participants had taken place during previous visits.

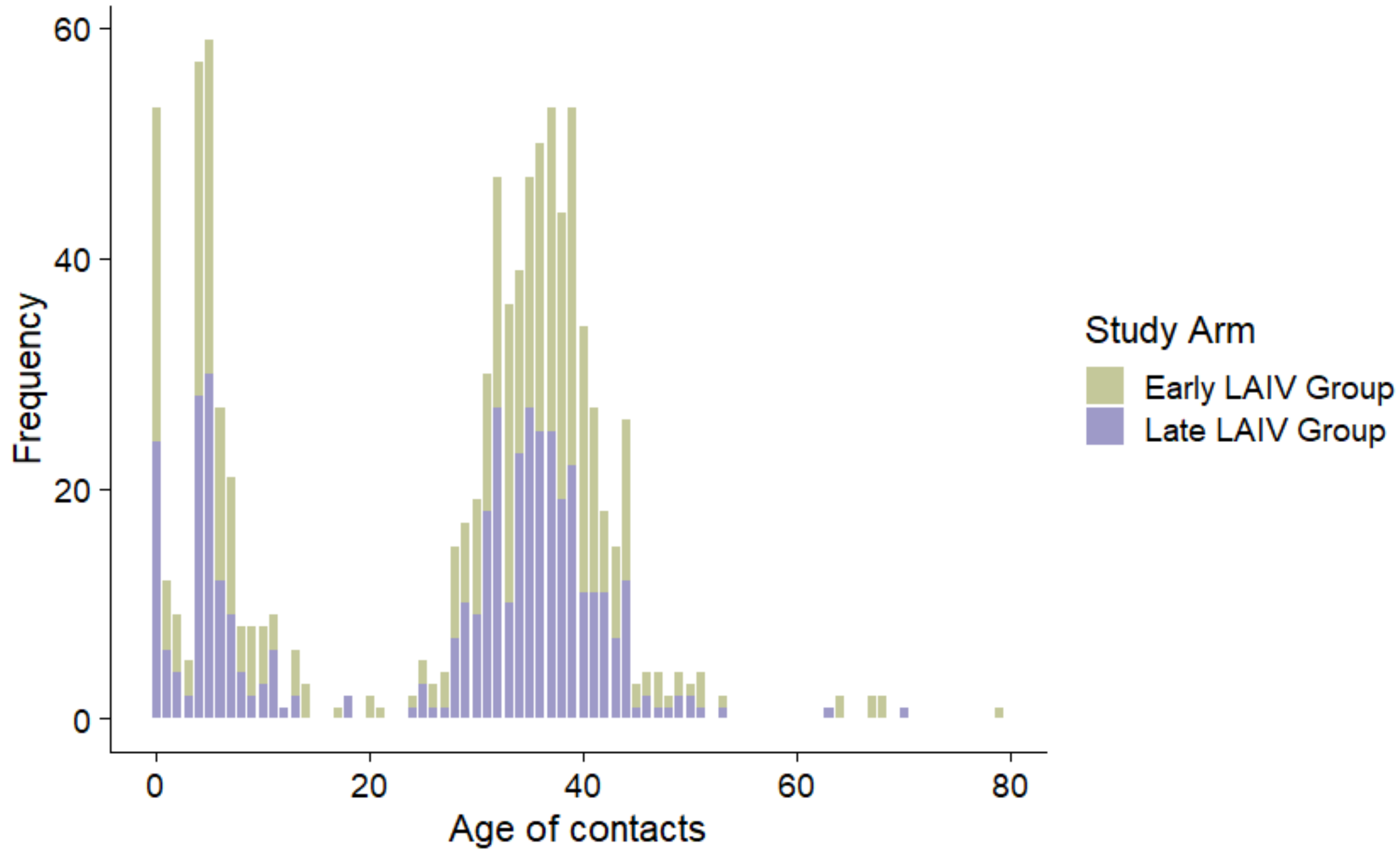

Figure A2: Age and frequency of the contacts of 2-year-old index children from the early and late (control) LAIV study arms. The majority of contacts were parents and caregivers of the index children.

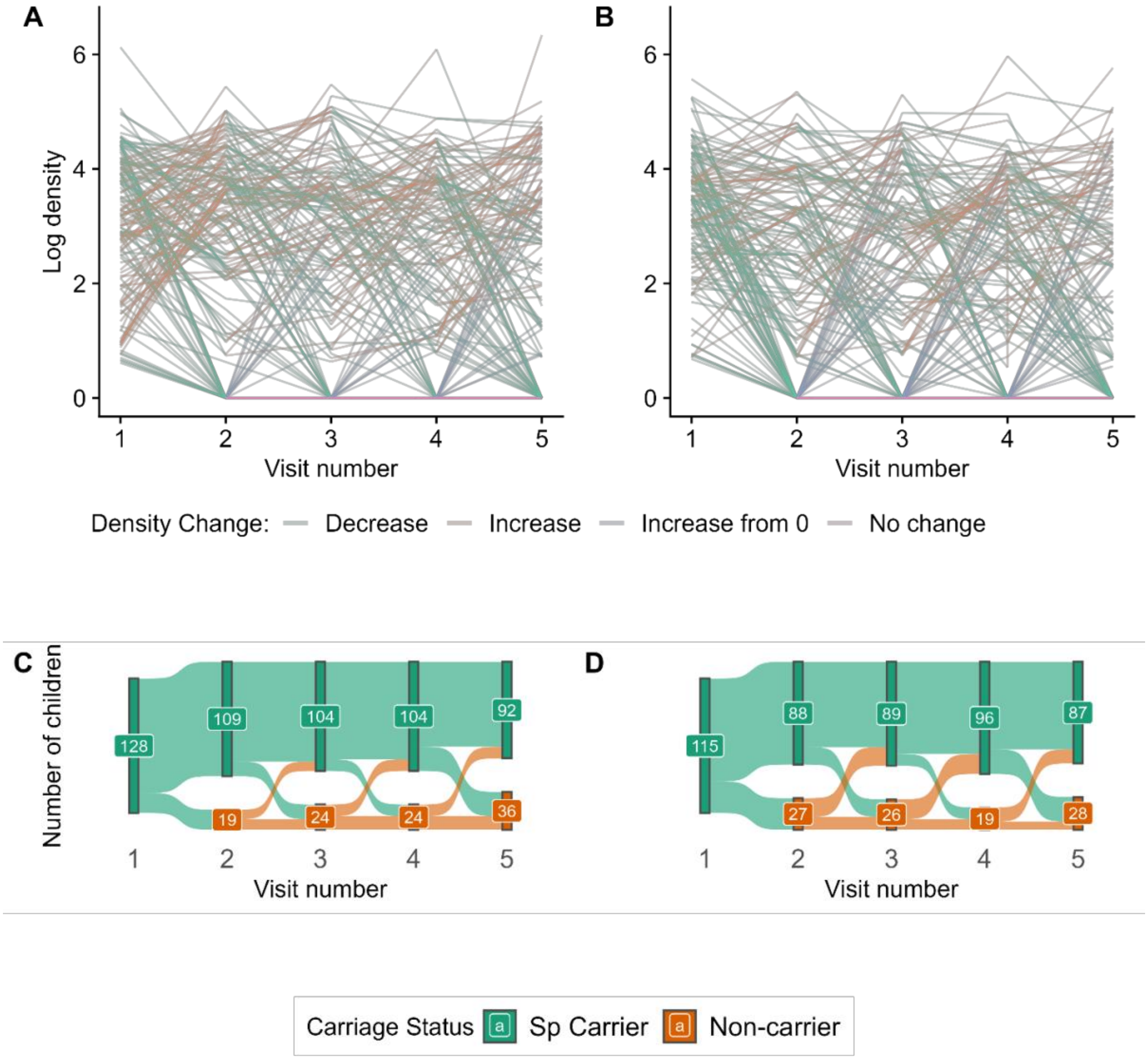

Figure A3: The dynamics of pneumococcal carriage density in visit 1-positive index children across all 5 visits in A. early LAIV group (n = 128) and B. control late LAIV group (n = 117). Green lines denote a decrease in density, blue lines denote an acquisition event (increase from 0) and red lines denote an increase. An increase in density was observed in 62/128 (48% 95%CI: 40-57%) of the index children who had received LAIV, at visit 2 14 days later, and in 32/117 (27% 95%CI: 20-36%) of those who had not received LAIV (Chi-square test p = 0.0007). Plots C and D are Sankey diagrams showing the number of index children who were Sp carriers and non-carriers across all 5 visits in the early and late LAIV groups respectively. Note: this was a per-protocol analysis including participants that had provided a sample at each study visit.

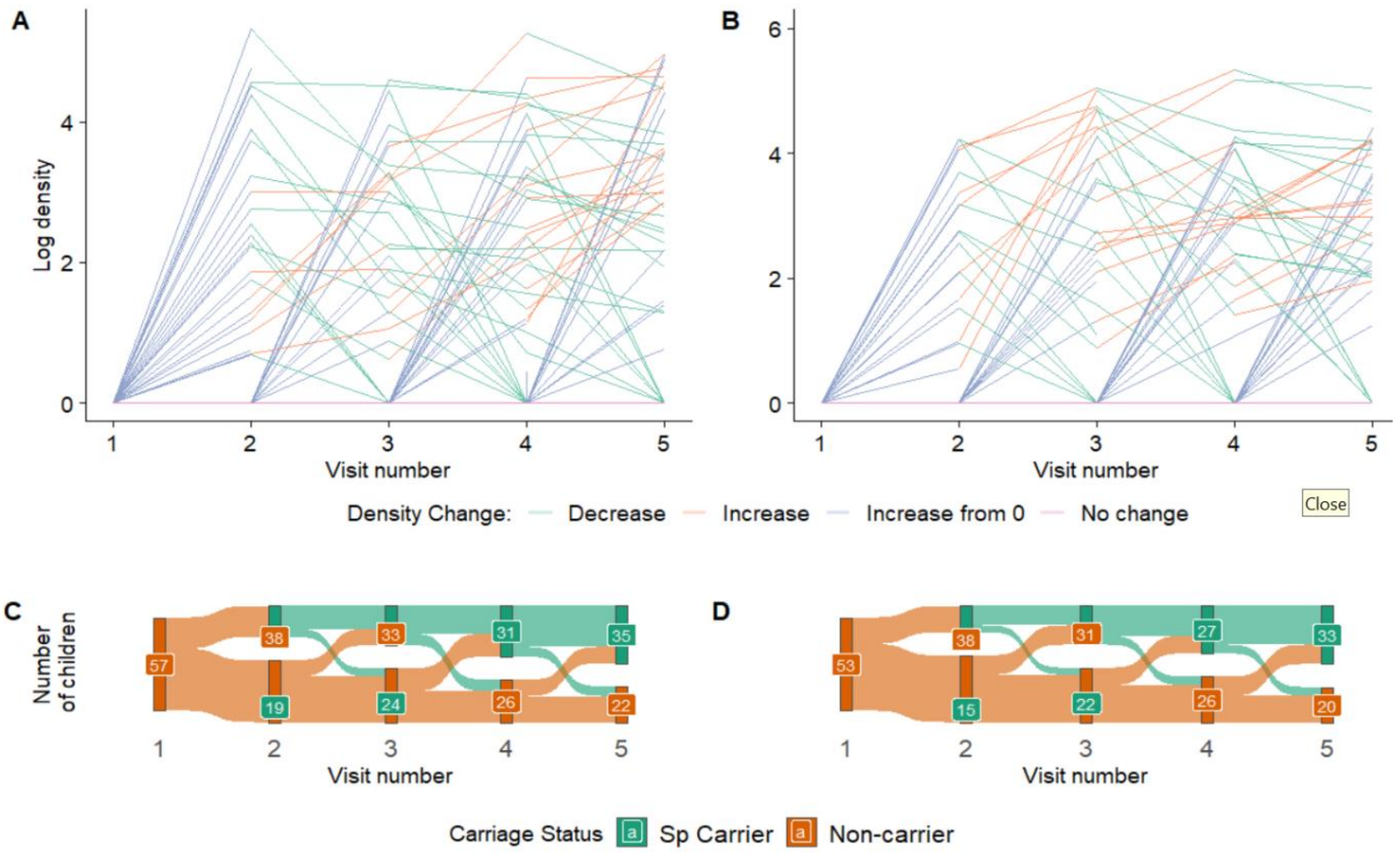

Figure A4: The dynamics of pneumococcal carriage density in visit 1-negative index children across all 5 visits in A. early LAIV group and B. control late LAIV group. Green lines denote a decrease in density, blue lines denote an acquisition event (increase from 0) and red lines denote an increase. Plots C and D are Sankey plots showing the number of index children who were Sp carriers and non-carriers across the 5 visits in the early and late LAIV groups respectively.

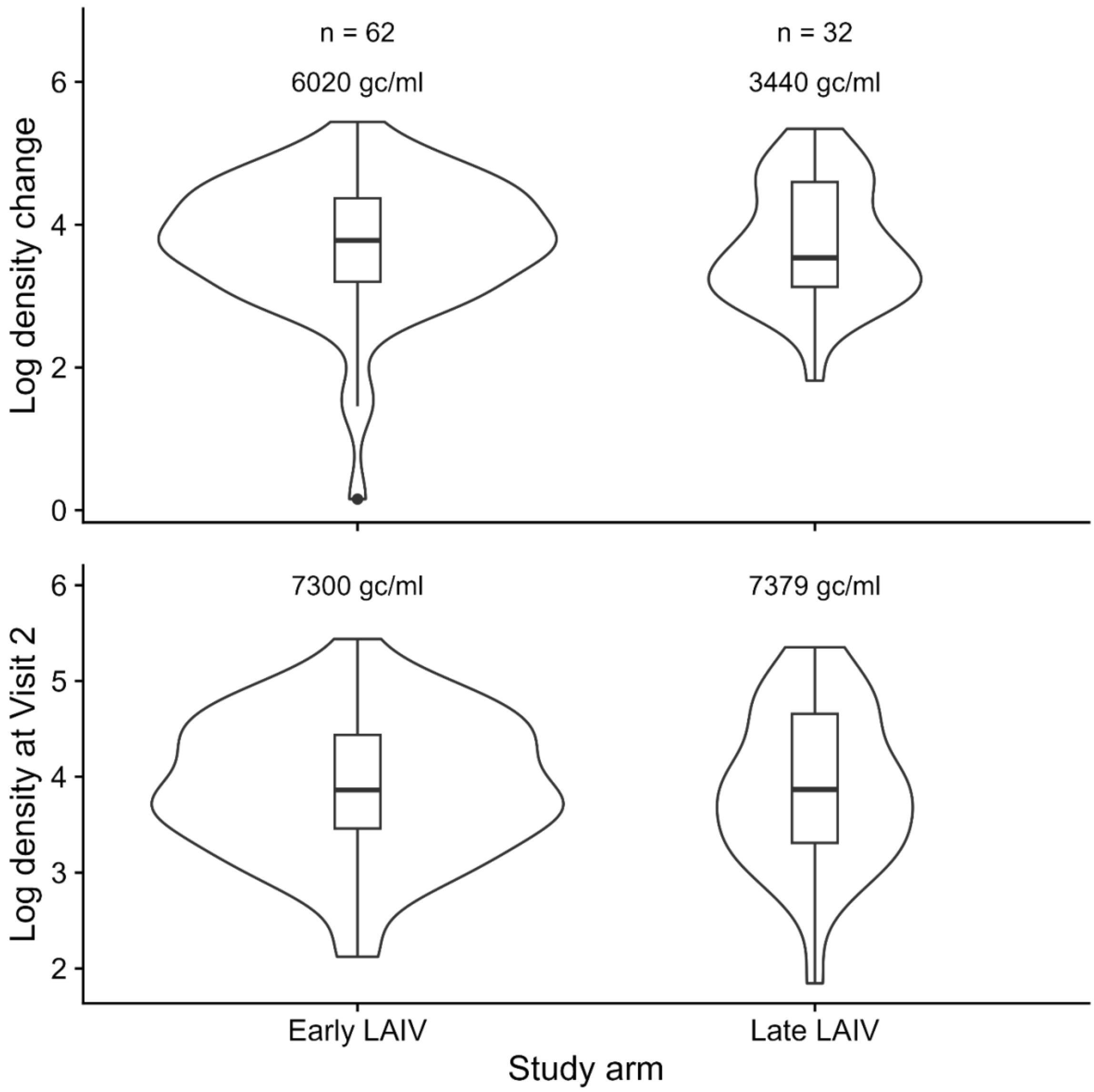

Figure A5: Change in pneumococcal carriage density in index children who were carriers at visit 1 and whose carriage density increased between visits 1 and 2. Children from the early LAIV group are shown on the right and control late LAIV group on the left. The median and interquartile ranges are shown width of the violin plots inside each violin. The numbers of children who carried pneumococcus at visit 1 and visit 2 and whose carriage density increased between these 2 visits in each group are represented by the width of the violin plots.

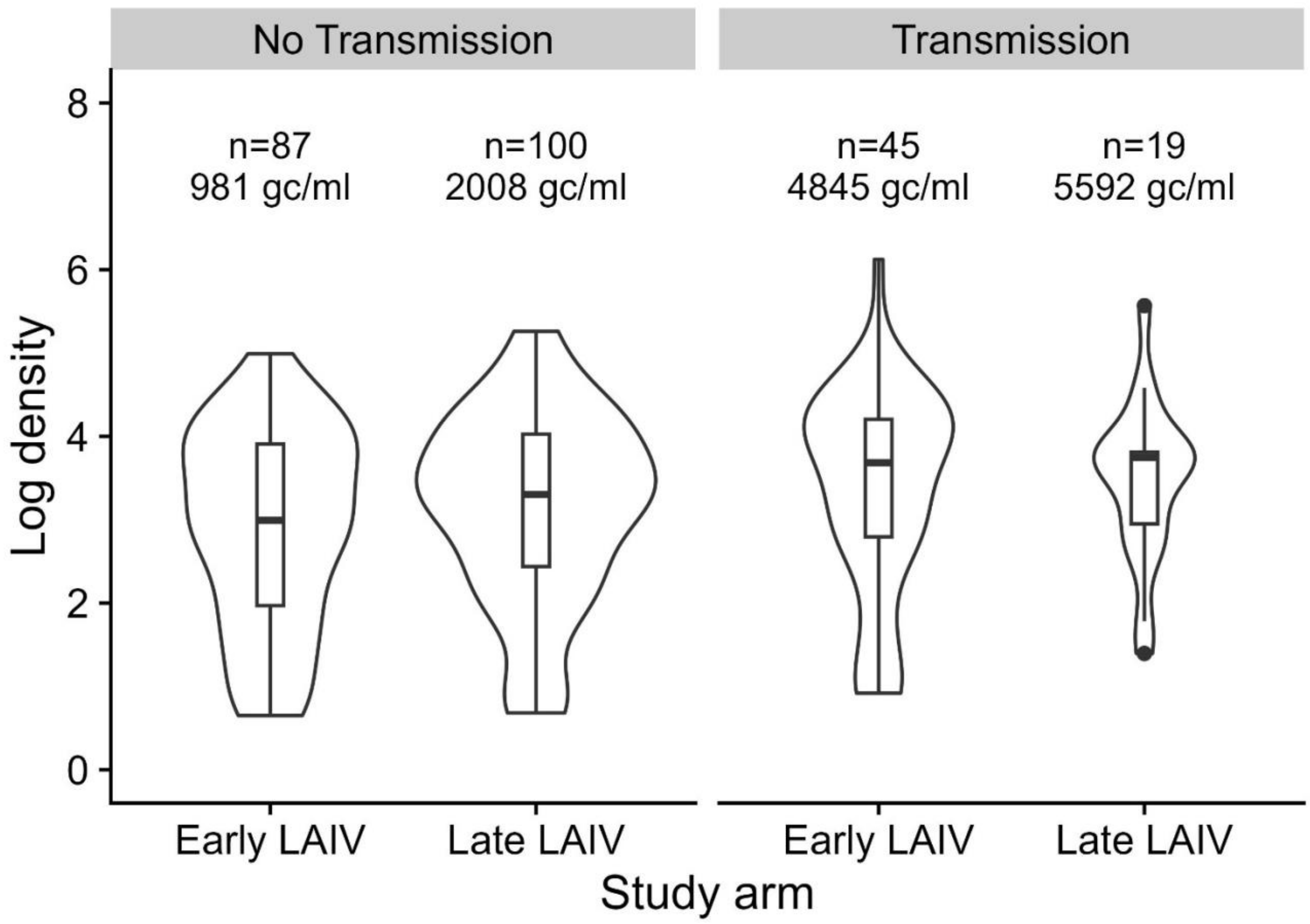

Figure A6: Carriage density of index children at visit 2 (14 days after LAIV in Early LAIV group) plotted according to whether an acquisition event had occurred or not and separated by study group. Note that we used the transform: log (Density+1) on the y-axis. The width of the plots represents the number of events. There was a significant statistical difference, Mann Whitney U-test, p = 0.029, between the carriage densities of index children where there had been a transmission event, n = 64, median density 5187 gc/ml compared with where no transmission event had been captured, n = 187, median density 1536 gc/ml. When this analysis was repeated but by study arm, a significant difference in pneumococcal densities was found in both groups: in the vaccinated early LAIV group, the median density of transmission-event positive index participants was 4845gc/ml (n = 45), compared with 981gc/ml (n = 87) in the transmission-event negative index, p = 0.026. In the unvaccinated late LAIV group; index pneumococcal carriage median density 5592gc/ml (n = 19) in transmission-event positive index compared with 2008gc/ml (n = 100), in transmission-event negative index, p = 0.28.

Inclusion/Exclusion criteria

Inclusion criteria

Index children

The study will recruit healthy male and female children whose 2nd birthday falls between 1st January and 31st August of the year of recruitment and who will be aged at least 2 years on the day of the first study visit, resident in and near the recruiting centres, who fulfil eligibility criteria ascertained following a health check and whose parent(s)/legal guardians offer signed informed consent to participate. Only index children who have at least two other people, either adults or children, resident in the house where they live and willing and able to take part (see below) will be enrolled. Children who live in more than one family home will be eligible if at least one home, where they spend at least half of their time (defined as half of their nights over the month preceding enrolment) has two other residents who are willing to participate.

Family contacts

The study will also recruit family contacts of index children who are willing to consent, or in the case of other children, whose parents are willing to consent (and for children old enough to understand the study, themselves assent) to completing study procedures and providing nose swabs and saliva samples.

Exclusion criteria

Index children

Exclusion criteria include all contra-indications in the vaccine licence, in addition:
· Children currently enrolled in another clinical trial or study which, in the opinion of the investigator might interfere with their likelihood of completing either or both studies or which might confound outcomes of either study. Families participating in the first year of the study who have another child of the right age and are otherwise eligible and who wish to participate again in the second year will be permitted to do so.
firmed or suspected primary or secondary immunodeficiency OR known to be severely immunosuppressed due to any condition or recent (in the last 6 months) therapy (e.g. leukaemia, HIV infection, organ or bone marrow transplant).
· Severely immunocompromised household member or close contact (because of potential for transmission of live attenuated virus)
· Receipt of immunosuppressants or immune modifying drugs including oral or parenteral steroids (at a dose equivalent of prednisolone >0.5mg/kg/day for more than 1 week within the 3 months prior to enrolment). Inhaled steroids are not a contraindication (although see below re asthma and wheezing).
· Current hospital-based management of asthma
· Previous receipt of, or intended immunisation with any other influenza vaccine(s).
· Children who are in risk groups for severe influenza (e.g. chronic respiratory (but for severe asthma see above), cardiac, renal, hepatic or neurological diseases and diabetes) can be enrolled and immunized in the study provided they are eligible to receive LAIV following the guidance published in the Green Book (www.gov.uk/government/uploads/system/uploads/attachment_data/file/456568/2904394_Green_Book_Chapter_19_v10_0.pdf).
· Children with other chronic, stable medical illnesses that do not result in immunosuppression and are not risk groups indicating receipt of seasonal flu vaccine (e.g. epilepsy, metabolic disorders) may

participate in the study, unless their condition or circumstances will in some way interfere with the completion of study procedures.
· Children receiving salicylate therapy (Aspirin)
· Previous anaphylactic reaction to any component of the nasal flu vaccine (including gelatine or gentamicin), or to eggs or egg proteins (e.g. ovalbumin).
· Any other condition or circumstances which, in the opinion of the investigator, might put the index subject or family members or research team members at risk or which might interfere with the likelihood of successful of study procedures and sampling per protocol or which might prevent the results obtained from contributing in any way with successful achievement of the study objectives.
Family contacts.
· Contacts resident in the same house at the index child for less than half of the time (defined, in principle, as half of their nights over the month preceding enrolment) will be excluded.

Temporary exclusion criteria for administration of nasal spray dose:

· Fever (>38.0°C) or history of high fever within the previous 24 hours or active wheezing at time of study visit.
· Immunisation within 3 days prior to immunization visit (21 days for any live vaccine), or plan to administer another vaccine within 7 days of study immunization (but see above re other flu vaccines). In such situations enrolment or study immunisation can be postponed where feasible.
· Acute Asthma: history of active wheezing/increased use of bronchodilators within 72 hours of study visit, currently taking a high dose of inhaled steroid (Budesonide >800mcg/day), or been on oral steroid treatment for asthma in previous 14 days
· A completely blocked/snotty nose that would inhibit effective nasal vaccination dose
· Any other medical condition or circumstances which, in the opinion of the investigator, will jeopardise the likelihood of safe or effective participation in the study.
3.3.5 Elimination criteria during the study for index 2-year-old subjects
Initiation of planned chronic administration (defined as more than 14 days) of immunosuppressants or other immune-modifying drugs during the study period (for corticosteroids, this will mean prednisolone or equivalent, ≥0.5mg/kg/day). Inhaled and topical steroids are not an elimination criterion.

Sample collection, nucleic acid extraction, qPCR set-up, and analysis

Detailed methodology including reagents, kits, and equipment are listed with supplier details (city, state/province, country) below:

- **QIAsymphony SP instrument** and **QIAgility pipetting robot**
  QIAGEN, Germantown, MD, USA
- **QIAsymphony DSP Virus/Pathogen Mini Extraction Kit** (QIAGEN 937036)
  QIAGEN, Germantown, MD, USA
- **Carrier RNA** and **AVE buffer** (1 µg/mL)
  QIAGEN, Germantown, MD, USA
- **T4/MS2 bacteriophage internal control** (300 µL)
  QIAGEN, Germantown, MD, USA
- **Elution Microtubes CL (24 × 96)** on cooling adapter
  QIAGEN, Germantown, MD, USA
- **Applied Biosystems ViiA 7™ Real-Time PCR System**
  Thermo Fisher Scientific, Waltham, MA, USA
- **QuantStudio 7 (QS7) Real-Time PCR System**
  Thermo Fisher Scientific, Waltham, MA, USA
- **TaqMan Fast Universal PCR Master Mix**
  Thermo Fisher Scientific, Waltham, MA, USA
- **MicroAmp Optical 384-well reaction plates**
  Thermo Fisher Scientific, Waltham, MA, USA
- ***Streptococcus pneumoniae* strain 6303 (ATCC)**
  via LGC Standards Laboratories, Teddington, UK

Nucleic acid (NA) extraction is required to make bacterial DNA available for qPCR analysis. The QIAsymphony SP instrument (QIAsymphony® QIAGEN, CA, USA) was used in conjunction with the QIAsymphony DSP Virus/Pathogen Mini Extraction Kit (QIAGEN 937036) and following the Virus Cellfree200 protocol. This is an automated process of NA extraction.

The automated extraction process within the QIAsymphony SP instrument can be simplified into the following steps taken directly from the manufacturer's instruction booklet (see booklet for schematic):

- Enzymatic bacterial cell lysis frees the NA.
- Silica-coated magnetic beads are added to bind the freed negatively charged NA.
- Insertion of magnetic rods to the NA-bound magnetic bead solution allows separation of the NA-bound-magnetic beads and subsequent transfer of NA into another tube for thorough washing with removal of unwanted material and then magnetic separation of NA from the beads (see booklet accompanying the QIAsymphony DNA Investigator kit).
- Total NAs are eluted into 110 µl elution buffer and dispensed into 96-well elution microtubes (QIAGEN, CA, USA) ready for use or storage.
- For each 340µl sample processed, a 110µl elution ready for qPCR is produced.

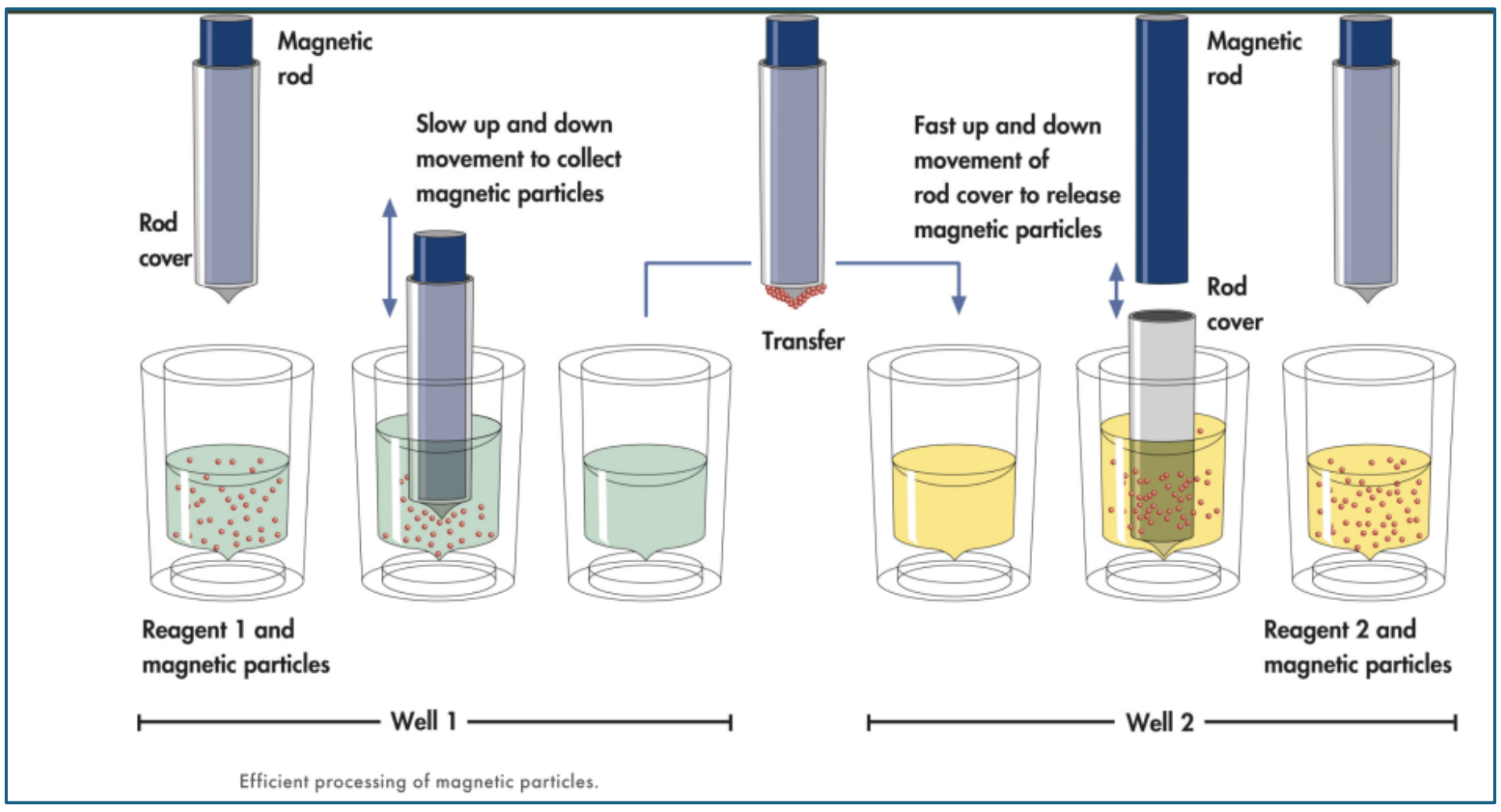

***Efficient processing of magnetic particle** (https://www.qiagen.com/us/products/human-id-and-forensics/investigator-solutions/qiasymphony-dna-investigator-kit)*

Participant samples were removed from the -70 ˚C freezer and thawed on ice. Each sample was vortexed for 30 seconds on maximum speed and 340µl was aliquoted into 2ml screwcap tubes. Each extraction run could process 96 samples (92 study samples and 4 negative controls). Negative controls consisted of 340µl of STGG. All tubes with barcoded sample identification inserts were placed into tube carrier racks and loaded into the QIAsymphony®. Other equipment and material required for the run was prepared and loaded into the QIAsymphony. This included:

- A QIAsymphony DSP Virus/Pathogen Mini Kit (QIAGEN 937036) cartridge
- Carrier RNA: 3.6ml cryotubes to aliquot carrier RNA 1350µg and AVE buffer (1µg/ml)
- Internal Control (IC): T4/MS2 bacteriophages 300ml, Carrier RNA 75ml, AVE buffer 3225ml
- A labelled extraction plate for the collection of nucleic acid (Elution Microtubes CL (24 x 96) (QIAGEN19588) loaded onto a 96-well-plate black cooling adapter which keeps the nucleic acid at =/<4°C after run completion
- Pipetting tips, sample-pre-cartridges and 8-rod-covers

Once the QIAsymphony was set-up, loaded and programmed, the process of NA extraction was completed without any further intervention. Each run took around 4 hours. The eluate plate was kept cool (0-4°C) until collection. On collection of the 96-well plate from the QIAsymphony, visual checks were carried out prior to elution cap strips being fitted (for contamination avoidance) and the plate was transferred to the -70°C freezer for storage.

Quantitative real-time polymerase chain reaction (qPCR)

Amplification and detection of DNA was carried out using the Applied Biosystems ViiA 7TM real time PCR system (Life Technologies, USA).Using the National Centre for Biotechnology Information (NCBI) database and the Basic Local Alignment Search Tool (BLAST), the specificity of the primers for the target species were assessed by the our group *in silico (Thors et al., 2016b)*.

Target	Gene	Forward Primer Sequence 5' – 3'	Reverse Primer Sequence 5' – 3'	[Dye]Probe Sequence 5' - 3'
S. pneumoniae	lytA	ACGCAATCTAGCAGATGAAGCA	TCGTGCGTTTTAATTCCAGCT	[Cy5]GCCGAAAACGCTTGATACAGGGAG[BHQ2]
T4	T4	GTTGAAAACGAATATCGGACGTAGT	CCTTGTAAGTACTGGGCAGTTTCTG	CY5-CAACGCGTTTACTCGTTCATCATTCCG-BHQ2

Extracted NA samples were thawed and centrifuged. The single plex MicroAmp optical 384-well reaction plate (Life Technologies, USA) required for qPCR was prepared using the QIAgility pipetting robot and software (QIAGEN, CA, USA). The pipetting robot was programmed to prepare wells containing a total volume 20µL containing:

- 10µL of TaqMan Fast Universal PCR Master mix
- 5µL of Primers and Probe (P/P) mix for the corresponding target (Sp or T4)
- 5µL of sample NA extract

The TaqMan Fast Universal PCR Master Mix (Thermo Fisher Scientific) was a comprehensive premix whose critical components included:

- AmpliTaq Fast DNA Polymerase: A thermostable enzyme to enable efficient amplification of DNA.
- Uracil-N glycosylase (UNG): An enzyme that mitigates contamination by degrading carryover DNA.
- dNTPs: A mixture of deoxynucleotide triphosphates, including dUTP, essential for DNA synthesis.
- ROX™ dye: A passive reference dye that facilitates accurate quantification in PCR assays.
- Optimized buffer: A formulation specifically tailored to enhance the performance of the PCR reaction.

This master mix ensures a streamlined approach for conducting real-time PCR, promoting reproducibility and reliability in quantitative nucleic acid analyses. Composition of P/P mixes for *lyt*A and T4 qPCR assays is shown below.

Target		Concentration (nM)
lytA	Forward primer (Spn-CDC-MCF)	300
	Probe (Spn-CDC-MCP Cy5)	100
	Reverse primer (Spn-CDC-MCR)	300
T4 (internal control)	Forward primer (T4-BV1F)	75
	Probe (T4-BVP1P Cy5)	25
	Reverse primer (T4-BV1R)	75

Internal control

T4 bacteriophage DNA was used as an internal amplification positive control to demonstrate that a negative qPCR sample result was neither due to failed NA extraction nor the presence of PCR inhibitors, but rather, a true reflection of the sample being negative for the tested target gene. The internal control

was prepared according to the extraction kit instructions with T4/MS2 bacteriophage Virus/Pathogen Mini Kit Public Health England.

Positive Controls

To confirm that the qPCR run had been set-up and was working correctly, and that a sample containing NA of the target gene would be detected if present, NA derived from serial dilutions of standard strain samples were run as positive controls with each qPCR run.

As a positive control for lytA detection, NA derived from 10-fold serial dilutions of ATCC American Type Culture Collection (ATCC) Sp strain 6303 (LGC Standards Laboratories, Teddington, UK) was used. For each 92-sample qPCR run, three positive control dilutions (1:2000, 1:20000 and 1:200000) were used.

As a positive control for ompJ detection (the target gene for Mc), NA derived from 10-fold serial dilutions of ATCC American Type Culture Collection (ATCC) Mc strain ATCC 25240 (LGC Standards Laboratories, Teddington, UK) was used. For each 92-sample qPCR run, three positive control dilutions (1:2000, 1:20000 and 1:200000) were used.

As a positive control for hdp detection (the target gene for Hi), NA derived from 10-fold serial dilutions of ATCC American Type Culture Collection (ATCC) Hi strain 10211 (LGC Standards Laboratories, Teddington, UK) was used. For each 92-sample qPCR run, three positive control dilutions (1:2000, 1:20000 and 1:200000) were used.

Negative Controls

DNA extracts of 4 STGG broth samples were used as negative reagent controls in each 92-sample qPCR sample run.

qPCR conditions

Using the QuantStudio 7 (QS7) Real-Time PCR System, qPCR was performed with the following conditions: there were two hold stages which are responsible for enzyme activation, one lasting 2 minutes at 50°C followed by a 20 second hold stage at 95°C. There were 50 cycles of 3 seconds each at 95°C followed by 1 minute at 60°C. This is the PCR stage, where DNA amplification and annealing of primers and probes to their specific sequences occurs. Samples with cycle threshold (Ct) values 35 and below were considered positive. ViiATM 7 Software version 1.3 (Applied Biosystems by Life Technologies, USA) was used for data analysis.

A UK Paediatric Vaccine Group study led by Bristol Children's Vaccine Centre, University of Bristol, UK

Study Protocol

Study Title: Evaluation of the relationship between pneumococcal colonisation density in 2-year-old children and rates of transmission to family contacts using live attenuated intranasal influenza vaccine as a probe. A randomised prospective step-wedge multicentre study.

Short Title: The Transmission Of Pneumococcus In Family Units Study

Acronym: TOP study

Study number/Internal reference number: 2779

ISRCTN registration number: xxxxxxxx

IRAS ID number: 227810

Protocol date and version number: version 1.12, dated 18/06/2018

Chief Investigator:

Prof. Adam Finn, BM, BCh, MA, PhD, FRCP, FRCPCH. adam.finn@bristol.ac.uk. Professor of Paediatrics, University of Bristol (UoB) and Honorary Consultant Paediatrician, Bristol Royal Hospital for Children, University Hospitals Bristol NHS Foundation Trust (UHB), Bristol, UK.

Co-Investigators:

Dr. Leon Danon, University of Exeter, UK

Prof. Margaret May, University of Bristol, UK

Dr. Peter Muir, Consultant Virologist, Public Health England (PHE) Virology Laboratory, Bristol, UK

Collaborating investigators:

Dr. Matthew Snape, University of Oxford, UK

Prof. Saul Faust, University of Southampton, UK

Prof. Paul Heath, St George's Hospital Medical School, London, UK

Dr. Stephen Hughes, Manchester Children's Hospital, UK

Sponsor:

University of Bristol, Research and Enterprise Development (RED), Senate House, Tyndall Avenue, Bristol, BS8 1TH, UK. Tel: +44 (0) 117 928 8676. Fax: +44 (0)117 929 8383.

Contents

1. List of abbreviations 4
2. Background information 5
2.1 Summary of the challenge we seek to address 5
2.2 The importance of the problem and potential value of the solution 6
2.3 Conceptual and methodological bases for the study 6
3. Study design 8
3.1 Explanation and justification 8
3.2 Objectives 10
3.2.1 Co-primary objectives 10
3.2.2 Secondary objectives 10
3.3 Participants and enrolment 10
3.3.1 Process for recruitment 10
3.3.2 Inclusion criteria 10
3.3.3 Exclusion criteria 10
3.3.4 Temporary exclusion criteria for administration of nasal spray dose 11
3.3.5 Elimination criteria during the study for index 2-year-old subjects 11
3.3.6 Informed consent 11
3.3.7 Withdrawal / discontinuation of participants from the study 12
3.4 Study Procedures 12
3.4.1 Randomisation 12
3.4.2 Health assessments 12
3.4.3 Immunisation, swabs and saliva samples 13
3.4.3.1 Nasal swabs 13
3.4.3.2 Saliva samples 13
3.4.3.3 Labelling of samples and laboratory blinding. 13
3.5 Benefits of participation 13
3.6 Retention of samples 14
3.7 Laboratory Sample Analysis 14
3.8 Definition of End of Trial 14
4. Study medication 14
4.1 Description, licensure and UK usage 14
4.2 Storage of vaccine 14
4.3 Administration 14
4.4 Safety Assessments 14
5. Safety Reporting 14
5.1 Definitions 15
5.1.1 Adverse Events (AE) 15

5.1.2 Serious Adverse Events (SAE) 15
5.1.3 Adverse Event Associated With the Use of the Drug 15
5.2 Reporting Procedures 16
5.2.1 All Adverse Events 16
5.2.2 Serious Adverse Events (SAEs) 16
5.2.3 Suspected Unexpected Serious Adverse Reactions (SUSARs) 16
6. Analysis and statistical analysis plan 16
6.1 Sample size calculation 16
6.2 Analysis population 17
6.3 Descriptive analysis 17
6.4 Analyses for study objectives 18
6.5 Statistical analysis of effects of flu vaccination on bacterial transmission 18
6.6 Missing data 18
6.7 Mathematical modelling of bacterial transmission dynamics 18
7. Direct Access to Source Data / Documents 19
7.1 Study Monitoring 19
7.1.1 Before the Study 19
7.1.2 During the Study 19
8. Study Conduct and Approvals 19
9. Data Handling 19
9.1 Data collection, recording and storage 19
9.2 Participant Confidentiality 20
10. Finance and Insurance 20
10.1 Finance 20
10.2 Indemnity and Insurance 20
11. Publication policy 20
12. References 21

1. List of abbreviations

AE	adverse event
C++	[name of statistics software package]
CI	Chief Investigator
CRF	Case Report Form
DNA	Deoxyribonucleic acid
EU	European Union
GCP	Good Clinical Practice
GLMM	Generalised Linear Mixed Models
GP	General Practitioner
Hi	*Haemophilus influenzae*
ICC	Intraclass correlation
ICH GCP	International Conference for Harmonisation of Good Clinical Practice
IMP	investigational medicinal product
LAIV	live attenuated nasal influenza vaccine
Mc	*Moraxella catarrhalis*
MHRA	Medicines & Healthcare products Regulatory Agency
Nm	*Neisseria meningitidis*
PCR	Polymerase chain reaction
PHE	Public Health England
PIS	Participant Information Sheet
qPCR	quantitative polymerase chain reaction
R	[name of statistics software package]
R&D	Research and Development
REC	Research Ethics Committee
RED	Research and Enterprise Development, University of Bristol
RNA	ribonucleic acid
Sa	*Staphylococcus aureus*
SAE	serious adverse event
Sp	*Streptococcus pneumoniae*
SSA	Site Specific Assessment
STATA	[name of statistics software package]
STGG	skimmed milk-tryptone-glycerol-glucose
SUSAR	Suspected Unexpected Serious Adverse Reaction
UHB	University Hospitals Bristol NHS Foundation Trust
UK	United Kingdom
UoB	University of Bristol
URT	upper respiratory tract

2. Background information

2.1 Summary of the challenge we seek to address

Many of the bacteria that commonly colonise the human upper respiratory tract (URT) are well known amongst clinicians as pathogens but pursue innocent lifestyles of colonisation and transmission unnoticed and unstudied nearly all of the time. Understanding this commensal biology has become important because it is now apparent that the public health impact of many of the universal vaccine programmes implemented worldwide are due as much or more to their indirect effects through reductions in colonisation and transmission within the broader population as through inducing specific immune responses protective against clinically significant disease in vaccine recipients – the latter being the mechanism evidence on which the development and licensure of all vaccines are currently based. A prime example of this is vaccines to prevent pneumococcal (Sp) disease. First generation polysaccharide Sp vaccines, although they induce mucosal antibody responses, have had little or no impact on carriage and transmission. By contrast, second generation conjugate Sp vaccines (PCVs), although they induce direct protection, cause powerful population-wide indirect effects that are increasingly shaping vaccine schedules and strategies, even though the microbiology and immunology behind colonisation and transmission and the way that vaccines alter them in humans are hardly understood at all. Likewise, much effort is being invested in discovery and development of third generation Sp vaccine antigens that will impact on transmission, without any clear strategies having been developed for testing such effects in the human population apart from introducing them and waiting to see what happens. This project seeks to take a critical first step in developing the Sp vaccine schedules and implementation strategies of the future.

Although data exist on rates of carriage of URT bacteria in different age groups measured as a binary variable (present or absent), we have only very recently described density profiles of colonisation [1-2]. It is widely accepted that, unlike those with low sputum bacillary numbers, patients with cavitatory pulmonary tuberculosis who have large numbers of organisms in their sputum are highly infectious. It therefore seems reasonable to hypothesise that individuals carrying much higher numbers of bacteria in their upper respiratory tracts are likely to be more infectious to others than those with fewer, but there are no experimental nor even observational data in humans that give any insight into the nature of the relationship between colonisation density and transmission rates to others. For example, although pre-school children attending a nursery are widely assumed to pass both viruses and bacteria to each other a lot, the basic reproduction number (R_o) values (the average number of secondary cases of an infection following a primary case in a completely susceptible population) for these infections in this setting are not known and so it is hard to predict accurately what proportion of them need to be rendered less infectious by vaccination (or how much less infectious and for how long) in order for circulation of a particular microbe within and beyond that nursery to be curtailed. That is the knowledge gap we are aiming to bridge with this study and those that will follow it. If we knew what impact a vaccine needs to have on the density and duration of carriage of Sp, or specific Sp serotypes, in order to reduce or eliminate transmission to others, we could design vaccines and vaccine schedules and test them against these objectives in children – in other words we could develop a microbiological correlate of protection against transmission, just as we currently have serological correlates of protection against invasive disease that enable us to avoid doing massive efficacy studies and to do small scale immunogenicity studies instead. Such information would also permit much more accurate dynamic transmission mathematical models to be developed to predict population effects of vaccines on particular bacterial species, strains and serotypes.

Empirical infection transmission studies can be conducted in animals where appropriate models exist[3] but the extrapolation of artificial experimental systems in another species to humans is problematic. Longitudinal observational studies in humans are feasible but the deductions about causality that can be made from them are limited and they often depend on the occurrence of chance events at the time of sampling[4]. Cluster randomised vaccine studies where acquisition of infection by unimmunised members of clusters is studied are a tool with which propagation of an infection within a defined group can be observed[5]. Infectious challenge studies are also in use to evaluate impact of vaccination upon acquisition of the target infection[6]. However, until now there has not been a viable, ethical and clinically relevant way to test how Sp colonisation density drives transmission in real life using a randomised interventional approach. Our recent demonstration, in a randomised trial, that administration of nasal

flu vaccine to healthy pre-school children induces increases in nasal colonisation density of *S. pneumoniae* (Sp) among carriers[7], provides an empirical tool with which to conduct just such a ground-breaking study.

This study will be the first of its kind and it cannot provide the last word on the subject. But its results will set the stage and inform the design of further studies including transmission to and between other age groups (including adults and the elderly) and settings (with different pneumococcal epidemiologies, vaccine usage histories and genetic and socioeconomic backgrounds). The approach will help explain many of the known, including some of the more unexpected observations about impact of high coverage PCV use on carriage of different serotypes and in different age groups.

We have assembled sufficient experimental and demographic data upon which to base our design to give it a high likelihood of scientific success. We have a group of experienced clinical, laboratory and epidemiological researchers and collaborators capable of overcoming the logistical challenges while maintaining efficient use of resources. Ultimately the results will lead to better strategic decision making about vaccine and vaccine programme design.

2.2 The importance of the problem and potential value of the solution

Since 1992, glycoconjugate bacterial vaccines against *Haemophilus influenzae* (Hi) type B, *Neisseria meningitidis*(Nm) groups A, C, W & Y and many serotypes of *Streptococcus pneumoniae*(Sp) have been introduced into many universal vaccine schedules. These vaccines were all licensed on the basis of their direct effects: inducing antibody responses that protect recipients from disease. Their effectiveness (and cost-effectiveness) in populations in all cases has turned out to depend as much or more upon their indirect effects: their ability to prevent bacterial transmission within the community and thus protect many of those who have not received vaccine or who are no longer protected by previous immunisation[8]. Recently a live intra-nasal influenza vaccine and a protein Nm group B vaccine have been introduced into the UK universal schedule, both for young children. In both cases the size and nature of indirect effects were not known at the time of introduction. In the former case, they form part of the cost-benefit case for vaccine use despite the uncertainty. In the latter, lack of information has prevented any decision to implement adolescent immunisation which, if such indirect effects exist, as they do for polysaccharide-conjugate meningococcal vaccines, might be a much more effective and cost-efficient strategy. Viral infections like flu increase the incidence of bacterial infections like pneumonia[9-10] so viral infections may be indirectly responsible for much bacterial morbidity and mortality and flu and other respiratory viral vaccines might prevent some bacterial as well as viral disease. Recent trends in UK meningococcal conjugate vaccine strategy reflect growing understanding of the need to target indirect effects. Similar ideas are beginning to be voiced about pneumococcal conjugate vaccination[11]. Better understanding of viral-bacterial interactions and bacterial propagation in the population and better prediction of the impact of both bacterial and viral vaccines upon transmission should result in the design of more effective and more efficient vaccine programmes, less disease, less antibiotic use and thus less emergence of antibiotic resistance. In order for correct policy decisions to be made and implementation strategies for new bacterial and viral vaccines to be correctly designed, we need to be able to predict indirect effects more accurately and so we need improved knowledge about how to measure and predict transmission and how vaccines affect transmission.

2.3 Conceptual and methodological bases for the study

Colonisation with upper respiratory bacteria in children is usually described as asymptomatic, although it may be associated with symptoms of rhinitis[12]. Although medical texts tend to focus upon the pathogenic potential of Sp and other respiratory bacterial species, they nearly always colonise and transmit between humans - especially young children and their contacts – without causing significant disease. The last decade has seen growth in publications about upper respiratory bacterial colonisation especially Sp since the recognition that the use of Sp conjugate vaccines in children at high coverage generally results in the disappearance of Sp serotypes contained in these vaccines and their replacement with others, overall colonisation rates remaining unchanged[13]. Initially carriage changes in immunised children were studied and subsequently population studies have been done to track the epidemiological impact of vaccine programmes in different settings[14-17].

Studies involve taking nasopharyngeal swabs and establishing the presence or absence of Sp by culture on selective agar plates, either shortly after the swab is obtained or after a period stored frozen in

enrichment (STGG) broth[18]. Formerly isolates were serotyped using agglutinating antisera, whereas now molecular techniques are generally used instead, including an innovative microarray approach capable of detecting several serotypes in a single sample and their relative abundance[19-20]. However absolute abundance of bacteria in these samples has received limited attention to date, partially because scoring colonies from cultures of dilutions of samples on agar plates is very labour intensive[12].

Availability of specific and accurate quantitative polymerase chain reaction (qPCR) assays to measure the presence and density of different serotypes and strains[21] should now change this. We have recently shown that different bacterial species can reliably be detected in DNA extracts from broth samples containing swabs using single gene real time qPCR assays[1]. It turns out that density of nasal colonisation with common bacterial species including Sp, Hi and *Moraxella catarrhalis*(Mc) in pre-school children can vary across 6 orders of magnitude with the majority of carriers residing in the upper 3 logs. It therefore seems reasonable to hypothesise (a) that changes in density of carriage alter transmission risk and (b) that the simple binary measurement of carriage as the predictor of infectiousness and herd protection maybe misleading e.g. a small reduction in carriage rate with larger falls in density might cause important effects on transmission that may be missed or under-estimated in studies evaluating effects of immunisation on carriage rates. Conversely elimination of large numbers of low density carriers with a smaller impact on high density carriage might over-estimate impact of vaccination indirect effects. However, by how much increases in density of carriage result in rises in rates of transmission is currently unknown.

There is a considerable literature on pathogenetic interactions between respiratory viruses and bacteria[22-23] much of which focuses particularly upon influenza and pneumonic infection with Sp and *S. aureus* (Sa) (recently reviewed in [24]). We have previously described age-independent associations between respiratory viral infection and both rates and density of Sp colonisation in healthy pre-school children[12]. Given that upper respiratory tract bacteria must both successfully colonise and transmit in order to survive in their niche, it is biologically plausible that such bacteria should evolve the means to sense viral infections or the host immune response to them and then to proliferate, alter their phenotype or both in order to profit from the transmission opportunity that virus-induced rhinitis affords them. Accordingly, if this effect exists and if one could mimic it using one or more clinically harmless viral infections, one could thereby potentially manipulate bacterial carriage density in a randomised interventional experiment in humans in order to study the relationship between bacterial colonisation density and transmission to close contacts. The licensure, availability and widespread use of cold adapted multivalent live attenuated nasal influenza vaccines for children from the age of 2 years affords the opportunity to undertake just such a study.

We have recently used qPCR detection and density estimation of colonisation by 4 bacterial species (Sp, Hi, Mc and Sa)*)* in healthy 2-4 year old children, in a prospective randomised step-wedge controlled trial to observe the effects of immunisation with live attenuated nasal influenza vaccine (LAIV)[7]. Carriage rates fell with increasing age for all bacterial species except Sa (which rose) and overall were Sp 69%, Hi 57%, Mc 78% and Sa 11%. Apart from a transient increase from 52% to 70% in Hi at 7 days (p=0.042), colonisation rates were not affected by flu vaccination. However, *S. pneumoniae* density was substantially higher in vaccine recipients (16,687 vs. 1935 gene copies per milliliter) 28 days after the first dose ($P < 0.001$). When all pre-vaccine samples were compared with all post-vaccine samples in a non-randomised analysis using generalized linear mixed models, adjusting for age, gender, presence of any smokers in the home, number of siblings, presence of respiratory viral nucleic acid in the samples, time and clustering effects of nurseries and repeated samples from the same individuals, there was evidence of an increase in the carriage rate of Hi (p=0.015) following vaccine and of higher carriage density of Sp (p=0.064) and of Mc (p=0.029) and weaker evidence for Hi (p=0.161). The numbers of Sa carriers were too few in this study to draw firm conclusions, although the observations made raise the possibility of an increase in carriage density evident at 28 days (visit 3 – V3) after immunisation, for this bacterial species as well[7]. The vaccine was in widespread use in North America in children from age 2 for over a decade and has been in the national programme in the UK since 2013 and there has been no safety signal reported, suggesting the changes we have observed are not associated with any clinically significant bacterial disease. This raises the possibility that LAIV (currently routinely used annually in 2-10-year-olds in the UK) could be used as a safe (and beneficial) experimental tool to increase bacterial carriage density in healthy children many of whom carry these bacterial species.

We now propose a randomised controlled study in 2-year-old children (referred to below as "index subjects") and their families to answer the question: by how much does increasing bacterial colonisation density increase transmission within family units?

3. Study design

3.1 Explanation and justification

The design of the study is shown schematically in the figure and discussed below. Measuring bacterial transmission from child to child within pre-school nurseries, the setting for our previous study, would be difficult, but detecting an increase in transmission from 2-year-old children to their household contacts, including siblings and parents, is expected to be feasible as baseline rates among these contacts are expected to be lower than in 2-year-olds, while close contact will be frequent. 2-year-olds will be studied as they will not previously have received LAIV and so will not have antecedent vaccine-induced immunity which might reduce LAIV effects on bacterial density. Wintertime peak density profiles of nasal carriage of Sp, Hi and Mc in this age group are shown in [1] indicating the very wide range of density observed and thus potential for detectable changes to occur. In the proposed study, changes in bacterial density (a continuous variable) in the 2-year-old index subjects represents exposure, while detection of new colonisation with Sp (and other bacterial species) in family contacts (a binary variable) represents occurrence of transmission. For the vertical analysis (between randomised groups) transmission will be deemed to have occurred when a family contact in whom pneumococcus is not detected at a study visit, *n*, has pneumococcus detected subsequently either at visit, *n+1* (e.g. if a contact is in whom Sp is not detected at the first visit, has Sp detected at visit 2). For the horizontal analysis, carriage rates at each study visit (proportion of family contacts in whom pneumococcus is detected) will be evaluated as a function of carriage density in the index child at the previous visit, and the time between visits.

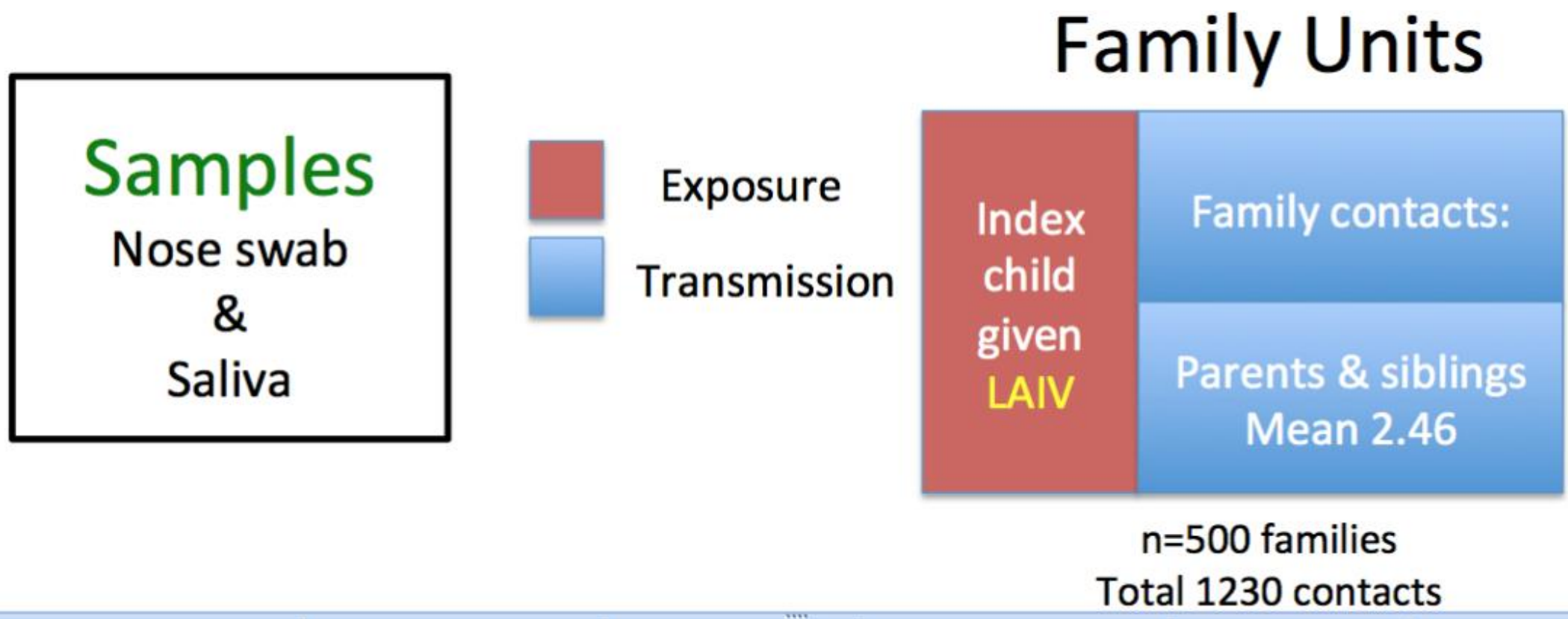

Randomised 1:1	V1 day 0	V2 day 14	V3 day 28	V4 day 42	V5 day 56
Group 1	LAIV Samples	Samples	Samples	Samples	Samples
Group 1	Samples	Samples	Samples	Samples	Samples
Group 2	Samples	Samples	LAIV Samples	Samples	Samples
Group 2	Samples	Samples	Samples	Samples	Samples

Figure 1. Study design outlined. Additionally, a follow up phone call will be made at 12-14 weeks. Windows for visits 2-5 are +/- 7 days

We propose to enrol 500 families over two sequential influenza seasons, across several UK centres, with a minimum of 2 resident contacts (adult or child) for each index subject (expected mean 2.46, so 1230 contacts). This number was chosen to give the study sufficient power for the primary and secondary objectives (see section 6 for power calculations). Families will be identified prior to the start of the enrolment period and contacted through local child health immunisation or other NHS databases (a well-established and ethics committee-approved mechanism of recruitment to vaccine trials). There will be 5 study home visits at 2 weekly intervals starting when vaccine becomes available in mid-September 2017 and ending in December 2017 and again Sept-Dec 2018. Home visits will ensure much higher on-

schedule retention and completion rates (critical for a study of this kind) by all participating family members than clinic-based visits where non-attendance is far more frequent. At the first study visit, the index 2-year-old subjects and all enrolled family members will have nasal swabs and samples of saliva collected and the index child will be randomised to receive LAIV either at the first or the third visit (the latter will occur in October-November, 1-2 months before seasonal influenza is normally seen in the UK). Siblings within the age range being offered routine LAIV immunisation (currently 2-10 years) will be offered vaccination at the last study visit by the study team and parents asked not to have them immunised through routine services in order to avoid any confounding effects. However, if a significant rise in influenza cases in the recruiting centres is unexpectedly noted before this time, vaccine will be offered without further delay. In order to be aware of the first signs of this, we will use the weekly reports produced by the Royal College of General Practitioners (GPs) which closely monitors rates of flu-like illness throughout the country all year round. No placebo nasal device is available, so the timing of immunisation will not be blinded to the parents or clinical research teams, but all samples will be barcoded and all laboratory analysis will be blinded until database lock., The index child and all family contacts enrolled will be asked to provide repeat swabs and saliva samples at each visit. Samples will be transported chilled to the laboratory and frozen for subsequent batched qPCR analysis for Sp, Hi and Mc using established protocols[1]. Social contact data will be collected using established survey methods[25] at all visits, to stratify contacts according to duration and proximity.

Studies from our group and others[12, 14, 17] indicate that older and younger siblings will have somewhat lower initial carriage rates of these three species than the 2-year-old index subjects. Recent studies from Oxford and the Netherlands[17, 26] report rates of carriage in parents of young children indicating that parents can be infected but that the large majority will be uncolonised at the start of this study. Using 2011 census data we can show that there will a sufficient surplus of households with a 2 year to 2 year ten month old child and at least two adult and/or child resident contacts across the research centres in 5 cities recruiting families to the study to permit 500 to be recruited even if the response rate is less than one in 50 (2%) (our previous single centre step wedge randomised LAIV swabbing study in nurseries enjoyed a 15% recruitment rate). We expect at least 730 (~60% of 1230) potentially informative contacts for the Sp objective while others will be informative for the other bacterial species under study (secondary objectives). Power calculations and statistical analysis plans are set out in detail in section 6 below. Consent will be sought at the time of enrolment from all subjects for retention and storage of residual samples beyond the end of the study. These will then be transferred to the Bristol Biobank and will be available for further analyses including serotyping (see below).

Appearance of Sp (or other bacterial species) in a family contact (our primary objective) may not necessarily represent transmission from the index 2-year-old index subject to the contact and could be confounded in several ways. These include sampling error (bacteria were previously present but not detected), acquisition from another family member or elsewhere or of a different strain of the same species. Our primary protection against these confounders is the randomised prospective laboratory-blinded nature of the study. In principle, such confounders should operate independently of vaccination and chance should dictate that they will be balanced between study groups so that any difference we observe between groups should be attributable to the randomised intervention. While it is conceivable that flu vaccination of the child may indirectly increase transmission between family contacts by onwards transmission of the vaccine viruses this is relatively unlikely, can be tested for and in some senses, can be regarded as part of the phenomenon we are aiming to study. Follow up studies may include confirmatory analyses of “transmission” sample pairs (by culturing lawns and isolates from samples from the index 2-year-old and the recipient contact of each event) in both study groups *post hoc* using serotyping microarray and whole genome sequencing, respectively, to evaluate in more detail the extent to which detection of the same bacterial species and strain in both child and contact occurred. Although doing this would not prove the direction of transmission, it would confirm to an extent the validity of the conclusions drawn from the original detection assays. Specifically, serotyping of Sp-positive (by lytA PCR) V1 swab and saliva samples from the index 2-year-old subjects would establish the serotype distribution in this population at the time of this study. This would in turn direct investigation of similarities/differences in transmission-density relationships in different prevalent serotypes and in different age groups (index child-child, index child-adult). An additional funding proposal-module has been provided for this extension to the core proposal – this will involve micro-array serotyping in collaboration with Jason Hinds at St George’s Hospital, London, UK.

3.2 Objectives

3.2.1 Co-primary objectives

1. To quantify the increase in transmission rates of Sp from 2-year-old index subjects to their family contacts following increases in density of carriage of Sp induced by administering LAIV.

2. To quantify the relationships between Sp carriage density among index children and contact duration[25] and probability of transmission.

3.2.2 Secondary objectives

The study will also quantify any increases in the probability of transmission of Hi and Mc following LAIV in a similar way.

3.3 Participants and enrolment

3.3.1 Process for recruitment

Parents of eligible children will be mailed an approved invitation letter about the study – access to information about the study will also be provided via the internet (study website). Families responding to this information by letter, email, other electronic messaging services or by phone will be provided with detailed study information, given an opportunity to ask any immediate questions they may have by telephone or at a preliminary consent study visit and then invited to schedule a first study visit. At this visit or these visits discussion and answering of questions, eligibility screening, informed consent, clinical assessment, randomisation, administration of the first vaccine dose (if randomised to early group) and collection of swab and saliva samples from the 2-year-old index subjects and family members will be done. Financial compensation for participating families to compensate for the time and inconvenience of participation in the study may be offered if/as approved by the ethics committee assessing the protocol.

Prior to randomisation, eligibility will be checked against inclusion and exclusion criteria defined in the protocol – see below. All study visits will be done in the family home or another location convenient for the family.

3.3.2 Inclusion criteria

Index children.

The study will recruit healthy male and female children whose 2nd birthday falls between 1st January and 31st August of the year of recruitment and who will be aged at least 2 years on the day of the first study visit, resident in and near the recruiting centres, who fulfil eligibility criteria ascertained following a health check and whose parent(s)/legal guardians offer signed informed consent to participate. Only index children who have at least two other people, either adults or children, resident in the house where they live and willing and able to take part (see below) will be enrolled. (Children who live in more than one family home will be eligible if at least one home, where they spend at least half of their time (defined as half of their nights over the month preceding enrolment) has two other residents who are willing to participate.)

Family contacts.

The study will also recruit family contacts of index children who are willing to consent, or in the case of other children, whose parents are willing to consent (and for children old enough to understand the study, themselves assent) to completing study procedures and providing nose swabs and saliva samples (as per figure 1).

3.3.3 Exclusion criteria

Index children. Exclusion criteria include all contra-indications in the vaccine licence, in addition:

- Children currently enrolled in another clinical trial or study which, in the opinion of the investigator might interfere with their likelihood of completing either or both studies or which might confound outcomes of either study. Families participating in the first year of the study who have another child of the right age and are otherwise eligible and who wish to participate again in the second year will be permitted to do so.

- Confirmed or suspected primary or secondary immunodeficiency OR known to be severely immunosuppressed due to any condition or recent (in the last 6 months) therapy (eg leukaemia, HIV infection, organ or bone marrow transplant).
- Severely immunocompromised household member or close contact (because of potential for transmission of live attenuated virus)
- Receipt of immunosuppressants or immune modifying drugs including oral or parenteral steroids (at a dose equivalent of prednisolone >0.5mg/kg/day for more than 1 week within the 3 months prior to enrolment). Inhaled steroids are not a contraindication (although see below re asthma and wheezing).
- Current hospital-based management of asthma
- Previous receipt of, or intended immunisation with any other influenza vaccine(s).
- Children who are in risk groups for severe influenza (e.g. chronic respiratory (but for severe asthma see above), cardiac, renal, hepatic or neurological diseases and diabetes) can be enrolled and immunized in the study provided they are eligible to receive LAIV following the guidance published in the Green Book (ref : www.gov.uk/government/uploads/system/uploads/attachment_data/file/456568/2904394_Green_Book_Chapter_19_v10_0.pdf).
- Children with other chronic, stable medical illnesses that do not result in immunosuppression and are not risk groups indicating receipt of seasonal flu vaccine (e.g. epilepsy, metabolic disorders) may participate in the study, unless their condition or circumstances will in some way interfere with the completion of study procedures.
- Children receiving salicylate therapy (Aspirin)
- Previous anaphylactic reaction to any component of the nasal flu vaccine (including gelatine or gentamicin), or to eggs or egg proteins (eg ovalbumin).
- Any other condition or circumstances which, in the opinion of the investigator, might put the index subject or family members or research team members at risk or which might interfere with the likelihood of successful of study procedures and sampling per protocol or which might prevent the results obtained from contributing in any way with successful achievement of the study objectives.

Family contacts.

- Contacts resident in the same house at the index child for less than half of the time (defined, in principle, as half of their nights over the month preceding enrolment) will be excluded.

3.3.4 Temporary exclusion criteria for administration of nasal spray dose

- Fever (>38.0°C) or history of high fever within the previous 24 hours or active wheezing at time of study visit.
- Immunisation within 3 days prior to immunization visit (21 days for any live vaccine), or plan to administer another vaccine within 7 days of study immunization (but see above re other flu vaccines). In such situations enrolment or study immunisation can be postponed where feasible.
- Acute Asthma: history of active wheezing/increased use of bronchodilators within 72 hours of study visit, currently taking a high dose of inhaled steroid (Budesonide >800mcg/day), or been on oral steroid treatment for asthma in previous 14 days
- A completely blocked/snotty nose that would inhibit effective nasal vaccination dose
- Any other medical condition or circumstances which, in the opinion of the investigator, will jeopardise the likelihood of safe or effective participation in the study.

3.3.5 Elimination criteria during the study for index 2-year-old subjects

Initiation of planned chronic administration (defined as more than 14 days) of immunosuppressants or other immune-modifying drugs during the study period (for corticosteroids, this will mean prednisolone or equivalent, ≥0.5mg/kg/day). Inhaled and topical steroids are not an elimination criterion.

3.3.6 Informed consent

Prior to enrolment and performance of any study procedures, informed consent will be obtained. Subject to ethical approval, consent to check previous influenza vaccine records may be obtained by phone prior to the first study visit. A parent or legal guardian of the index subject and other child

participants in the family must personally sign and date an appropriate informed consent form for each child. Signed assent forms will also be obtained from older children where appropriate. All family members who agree to participate who are aged 16 years or older must personally sign an appropriate informed consent form themselves. Signed informed consent forms will be countersigned and dated by the trained clinical research team member present when appropriate.

Appropriate written participant information will be presented to the parent(s), legal guardian and other participants which explain the exact nature of the study including all study procedures, the implications and constraints of the protocol, the known side effects and any risks involved in taking part. Such information will have been provided to the family at least 24 hours before the first formal (randomisation) study visit. At the time of consent, all those involved will have been given a chance to ask questions and have them satisfactorily answered before giving consent. It will be clearly stated that any participant or parent/legal guardian is free to withdraw themselves and/or his/her child from the study at any time without prejudice to future care and with no obligation to give the reason for withdrawal.

Information will include details of proposals to take and to keep samples for additional infection and immunity-related research studies which may include evaluation both of microbes present and the host and host immune responses and genetic studies of both the microbiome and host. All those giving consent will be able to participate in the study either with or without giving consent to any such additional/follow on studies and associated long-term sample storage. Explicit separate optional consent for any subsequent host genetic research on suitably anonymised samples will be open to those giving consent.

3.3.7 Withdrawal / discontinuation of participants from the study

Each participant and the parent(s)/legal guardian of all children participating in the study may withdraw (them) from the study at any time. In addition, the investigator may withdraw a participant from the study at any time if s/he considers it necessary for any reason including, ineligibility arising or becoming apparent during the study, significant protocol deviation, an adverse event which requires discontinuation of the study or which results in inability to continue to comply with study procedures or consent withdrawal.

If the participant is withdrawn from the study the samples and any data already collected may be included in the analysis provided consent to do so is not withdrawn.

The reason for any withdrawals will be recorded if parents/guardians/participants are willing to disclose them. If the participant is withdrawn due to an adverse event, the investigator will arrange for follow-up visits or telephone calls until the adverse event has resolved or stabilised.

3.4 Study Procedures

3.4.1 Randomisation

After informed consent has been obtained, the index subject will be given a study number, which will be used for randomisation. Children will be randomised into one of two groups to receive LAIV at different times (see figure 1).

Individual index subjects will be randomised with equal (1:1) allocation between treatment arms. The allocation will be ascertained using a single central allocation service/computerised randomisation for all sites.

3.4.2 Health assessments

Timings of study visits and procedures are shown in figure 1. At visit 1, eligibility checks will be carried out prior to sampling and immunisation of the index child (if applicable). Relevant information will be recorded on a standardised case report form, including demographic information such as family structure. The index child and all family contacts being studied will also have any current or recent antibiotic use and a rhinitis score recorded at each visit.

At all subsequent visits further health checks will be completed by a member of the study team for the index child, including a score for current rhinitis and nasal congestion symptoms and history of any

other symptoms and of medicines received between visits. Rhinitis scores for all family contacts being studied will also be recorded for the day of each visit.

3.4.3 Immunisation, swabs and saliva samples

The administration of vaccine and the taking of nasal swabs and saliva samples will be conducted at home visits by suitably qualified and trained clinical research team members following standard operating procedures.

All index subjects will receive a single dose of LAIV either at the first or at the third study visit (figure 1). Any siblings within the relevant age range (2-10 years) will receive the vaccine at the final study visit.

All study subjects (index children and family contacts) will provide 5 nasal swabs and 5 saliva samples (figure 1 above). If they are present at study visits samples will be taken then by research team members. Family members unable to attend study visits may also provide samples by taking them themselves or from each other, labelling them and storing them in the hours prior to the visit, following appropriate training and instruction and provision of necessary equipment by the study team. If necessary additional visits to obtain samples may be scheduled, when feasible. The duration of follow up will be up to 14 weeks from the first study visit, including a follow up telephone call approximately 1 month after the last visit. Any vaccinated children who have ongoing adverse events at this time will be further followed up until resolution.

3.4.3.1 Nasal swabs

A sterile nasal brush-swab with a 2cm long head will be carefully introduced into the subject’s nose inferiorly and horizontally towards the nasopharyngeal space until it meets slight resistance or the subject resists or objects, when it will quickly be removed using a twisting action along the axis of the shaft of the swab. The swab head will then be cut off, using sterile scissors and introduced into a 2ml container with 1.5 ml skimmed milk-tryptone-glycerol-glucose (STGG) broth [24]. This will then be refrigerated / kept on ice until returned to the laboratory (usually within 3-4h) where it will be vortexed and frozen at minus 70-80°C until analysis.

When feasible, a second swab or saliva sample may be obtained at any visit from any study participant by a study team member and placed into a container of RNA-preservation medium to permit additional transcriptomic studies (subject to consent – see above). These samples will be refrigerated and transferred to the lab as above, vortexed, stored at 4°C overnight, and then frozen and minus 70-80°C until analysis.

3.4.3.2 Saliva samples

Depending on age, the former method being employed in general in younger subjects, saliva samples will be obtained either using sterile sponges attached to sticks which, once saturated in the mouth, will be transferred into specimen tubes containing 1ml of STGG (or RNA-preservation medium – see above). Once in the lab, the saliva will be squeezed from the sponge and then stored and handled as above. Alternatively, older children and adults will be asked to spit/drool into a funnel attached to a specimen container, samples thereafter being handled in the same way.

3.4.3.3 Labelling of samples and laboratory blinding.

All samples will be labelled with a barcode label. A code list linking the bar codes to the subject and visit numbers and sample type will be kept securely in the clinical research office in a separate location from the laboratory to permit code breaking once primary lab analyses are complete and the database locked. All laboratory analyses will thus be performed blind.

3.5 Benefits of participation

All index subjects and siblings within the appropriate age range will receive the live current seasonal influenza vaccine as per the UK national schedule. This confers no additional benefit to routine practice at present. Subjects will have a health check prior to enrolment and immunisation at a home visit. Families will receive financial compensation in the form of vouchers of £15 per family per sampling visit (amounting to a total of £75 for all 5 visits) for the time and inconvenience involved in participation, subject to ethics approval. Families will be able to contact the study team throughout their participation to report any concerns and obtain appropriate advice.

3.6 Retention of samples
Where appropriate consent is offered, residual samples will be retained after the end of the study in the Bristol Biobank to permit further research to be undertaken. The regulations and approvals of the Biobank permit samples to be retained indefinitely.

3.7 Laboratory Sample Analysis
Bacterial detection and quantification for Sp, Hi and Mc will be performed using real-time quantitative PCR assays as previously reported[1, 7]. Lawn cultures and microarray serotyping will be done as previously described[27-28].

3.8 Definition of End of Trial
The end of the trial, following completion of all study visits and phone calls, is the date of completion of conduct of all study assays, completion of analysis of all data, database lock and submission of the final report.

4. Study medication

4.1 Description, licensure and UK usage
The vaccine is a tetravalent nasal live attenuated cold-adapted influenza vaccine (Fluenz tetra®, MedImmune, Astra-Zeneca). The trivalent vaccine (2 influenza A, 1 influenza B strains) was licensed in the EU in October 2010 for children aged 2-17 years. The tetravalent vaccine was licensed 2014. The trivalent vaccine was offered to all 2 and 3-year-olds in the UK as a single dose in the winter of 2013-4. In subsequent years, the upper age limit was progressively raised reaching 10 years in 2018-19 and the tetravalent vaccine has been used since this became available.

4.2 Storage of vaccine
Study medication will be stored and dispensed by the clinical research pharmacy departments in the participating centres in accordance with Good Clinical Practice and Good Manufacturing Practice.

4.3 Administration
Fluenz tetra® is an intranasal vaccine which will be administered using a one dose spray device into each nostril. The vaccine will be administered by an appropriately clinically qualified and trained member (nurse or doctor) of the study team.

Dosage: One dose of 0.2 ml LAIV (Fluenz tetra®) will be intranasally administered (0.1ml in each nostril) to the index subject either at study visit 1 or at study visit 3 according to randomisation. Siblings within the appropriate age range will receive the vaccine at the final study visit (visit 5) or as soon as possible if rapid rises in flu circulation are observed in the local community before visit 5.

A single dose of LAIV is currently offered to all children from age 2 years each year in the UK. The upper age limit for immunisation has been being increased year on year and most recently is 10 years.

4.4 Safety Assessments
Standard immunisation practice will be observed. All children will be monitored for a period of up to 15 minutes after the administration of the vaccine. In case of an allergic reaction the study doctor or nurse will evaluate the need for medical intervention (in the form of adrenaline or anti-histamine medication). In the case of a severe or anaphylactic reaction, adrenaline (1:1000) will be administered intramuscularly and appropriate medical assessment and treatment organised, usually by emergency transport of the child to the local emergency department for children for further treatment and observation. After discharge, the parents of any such child will be contacted at least weekly until the condition is stable and the GP will be informed with a written report.

5. Safety Reporting
This is a post licensure study without safety objectives. Nevertheless, adverse events will be recorded.

5.1 Definitions

5.1.1 Adverse Events (AE)

AEs are defined as any untoward medical occurrence in a clinical study participant. An AE does not necessarily have to have a causal relationship with the study treatment. An AE can therefore arise from any unfavourable sign (including an abnormal finding), symptom or disease temporally associated with the use of a medicinal (investigational) product, whether or not considered to be related to the medicinal (investigational) product. (International Conference on Harmonisation [ICH] definition). This includes any occurrence that is new in onset or aggravated in severity or frequency from the baseline condition, or abnormal results of diagnostic procedures, including laboratory test abnormalities.

All ascertained AEs will be recorded in the Case Report Form (CRF) for the duration of the participant's involvement in the study.

Standard procedures for notification of AEs and SAEs as followed for all licensed vaccines that are in routine use will be used.

5.1.2 Serious Adverse Events (SAE)

A SAE is defined by ICH as any untoward medical occurrence that at any dose of the study medication meets any of the following conditions (potentially relevant to this study):

i. **Results in the death of the participant**

ii. **Is life-threatening**
The term "life-threatening" refers to an event in which the participant was at risk of death at the time of the event; it does not refer to an event which hypothetically might have caused death if it were more severe.

iii. **Requires inpatient hospitalisation or prolongation of existing hospitalisation**
For any event that may not be immediately life-threatening or result in death or hospitalisation, but may jeopardise the participant or may require intervention to prevent one of these outcomes (for example, oedema or allergic bronchospasm that required intensive treatment at home, blood dyscrasia or convulsions), the CI should exercise his/her scientific and medical judgement to decide whether or not such an event requires expedited reporting to UH Bristol (who acts on behalf of the Sponsor in these instances).

iv. **Results in persistent or significant disability / incapacity**
Any event that seriously disrupts the ability of the participant to lead a normal life, in other words leads to a persistent or permanent significant change, deterioration, injury or perturbation of the participant's body functions or structure, physical activity and/or quality of life.

5.1.3 Adverse Event Associated With the Use of the Drug

An AE is considered to be associated with the use of the drug if the attribution is possible, probable, or very likely by the definitions listed below.

- **Not related:** An AE that is not related to the use of the drug.
- **Doubtful:** An AE for which an alternative explanation is more likely, e.g., concomitant drug(s), concomitant disease(s), or the relationship in time suggests that a causal relationship is unlikely.
- **Possible**: An AE that might be due to the use of the drug and for which an alternative explanation, e.g., concomitant drug(s), concomitant disease(s), is inconclusive. The relationship in time is reasonable and therefore, the causal relationship cannot be excluded.
- **Probable***:* An AE that might be due to the use of the drug. The relationship in time is suggestive (e.g., confirmed by IMP withdrawal). An alternative explanation is less likely, e.g., concomitant drug(s), concomitant disease(s).
- **Very likely:** An AE that is listed as a possible adverse reaction and cannot be reasonably explained by an alternative explanation, e.g., concomitant drug(s), concomitant disease(s). The relationship in time is very suggestive (e.g., it is confirmed by IMP withdrawal and re-introduction).

5.2 Reporting Procedures

5.2.1 All Adverse Events

As the LAIV is a licenced vaccine, no active solicitation of adverse events will occur. However, reported AEs will be documented from the time a signed and dated informed consent form is obtained until completion of the last study-related procedure. Those occurrences meeting the definition of SAEs must be reported using the Serious Adverse Event Form (available from the study team in Bristol), including SAEs spontaneously reported to the Investigator within 30 days after the participant has completed the study (including post study follow-up). UHB, on behalf of the Sponsor, will evaluate any safety information that is spontaneously reported by a CI beyond the time frame specified in the protocol.

All AEs, regardless of seriousness, severity, or presumed relationship to study drug, must be recorded in the source document and the CRF, together with any measures taken. CIs must record in the CRF their opinion concerning the relationship of the adverse event to study therapy. UHB, on behalf of the Sponsor, assumes responsibility for appropriate reporting of adverse events to the regulatory authorities.

Mild nasal congestion, fever with a duration of ≤ 1 day, and/or mild irritation should not be reported as an adverse event.

5.2.2 Serious Adverse Events (SAEs)

All SAEs must be reported to the UHB contact by investigational staff within 24 hours of their knowledge of the event.

All SAEs that have not resolved by the end of the study, or that have not resolved upon discontinuation of the participant's participation in the study, must be followed until any of the following occurs:

- the event resolves
- the event stabilizes
- the event returns to baseline, if a baseline value is available
- the event can be attributed to agents other than the study drug or to factors unrelated to study conduct
- when it becomes unlikely that any additional information can be obtained (participant or health care practitioner refusal to provide additional information, lost to follow-up after demonstration of due diligence with follow-up efforts)

The death of a participant is considered an SAE, as is any event requiring hospitalization (or prolongation of hospitalization) that occurs during the course of a participant's participation. Exceptions to this are hospitalizations for:

- social reasons in absence of an adverse event
- surgery or procedure planned before entry into the study (must be documented in the CRF)

5.2.3 Suspected Unexpected Serious Adverse Reactions (SUSARs)

All relevant information about a SUSAR which occurs during the course of the study and is fatal or life-threatening will be reported as soon as possible to the MHRA and the relevant ethics committee by UHB, on behalf of the Sponsor. The expectedness of an adverse event will be determined by whether or not it is listed in the Summary of Product Characteristics.

6. Analysis and statistical analysis plan

6.1 Sample size calculation

In our previous study we observed increases in bacterial density of nasal colonisation amongst carriers of Sp, Hi and Mc in children following vaccination with LAIV (see section 2.2 above). Here we propose to measure rates of Sp transmission by measuring carriage rates in contacts of children vaccinated with LAIV, compared with carriage rates in contacts of children not vaccinated with LAIV. This will allow us to quantify the increase in rates of Sp transmission resulting from the effects of the vaccine. The effect size, our primary objective, should clarify the relationship between carriage density in the vaccinated children

and their transmission potential. Likely effect sizes are not available from the literature because this has not been studied previously. However, we do know, from the literature and our previous research, that 60-70% of pre-school children are carriers of Sp, so that we expect more than half of the overall sample of families will be informative for the primary objective. At baseline, we would expect more than 15% of household contacts (the likely principal contacts of the children) to be carriers of Sp (with a carriage rate of at least 9% in parents, and up to 60% in other children in the household)[26]. Detection of a 2.0-fold rise in transmission (e.g. a comparison of 15% Sp carriage in contacts in the control arm compared to 30% carriage in intervention arm) with 90% power would require a sample size of 260 household contacts of children carrying Sp at the outset per study arm i.e. 520 contacts of carriers in total (assuming a 50% Sp carriage rate and a normal approximation to the binomial distribution).

For the secondary objectives, Hi and Mc, carriage rates in the population are different, modifying our expectations. For Hi, we expect 57-76% of children and 30% of parents to be carriers (our data and [17]). In this setting, with 90% power and 280 household contacts or carriers per study arm (total sample size 560), we would be able to detect an effect size as low as 1.7-fold. A 1.5-fold rise would be detectable at 80% power, with a total sample size 548. For Mc, we expect a higher rate of carriage in children (78-80%) and 22% in their contacts (our data and [17]). In this setting, we would require a total sample size of 570 household contacts of carriers for detecting a 1.9-fold increase with 90% power, and 524 household contacts of carriers to detect a 1.7-fold increase with 80% power.

We estimated the expected number of social contacts of children by considering household sizes from the 2011 census for the five areas that we propose to recruit from. We restricted the calculation to those households with children aged between 2 years and 2 years 10 months (assuming a constant birth rate), with at least 2 contacts for the children i.e. families with one child and at least 2 adults, at least 2 children and at least 2 adults, and one adult and at least 2 children. Using a conservative assumption that those households listed as being comprised of at least 2 adults or children in the census are comprised of only 2 adults or children, we can show from the census data that each child has on average 2.46 contacts, so that 212 households would be required to obtain 520 household contacts. If we allow for half of approached households to be non-informative (i.e. children to be vaccinated are not carriers of Sp at the outset), we would need 424 households to achieve the required 520 contacts for the primary objective. Assessing this across 5 study sites, 29,706 households fulfil these criteria, and, as only 1.4% of available household would need to be recruited, there is a considerable surplus of households potentially available for this study.

Accordingly, a total study size of 500 children and their families, yielding 1230 contacts would be sufficient to detect the effects for both the primary and secondary objectives, allowing for 20% drop out in a population with at least a 60% carriage rate in the index child since this would yield 590 contacts of informative index cases (500x2.46x0.8x0.6). Detection of larger-fold rises, which are certainly plausible, would require fewer in each group or have greater power. We have not allowed for clustering within families of index cases in the above calculations as it is difficult to estimate what the correlation would be without performing the study. However, if we assume a conventional intra-class correlation coefficient of 0.05, then the design effect would be 1.073 and the required number of household contacts for the primary objective would increase from 520 to 558. In fact, the ICC could increase to 0.09 before the study power would decrease below 90%.

6.2 Analysis population

Analyses will include all data points collected in the study from persons from whom we gain informed consent.

6.3 Descriptive analysis

Baseline characteristics of the study population will be summarised separately for each study centre, and for the study as a whole.

For categorical variables, the number and percentage of participants within each category will be presented. For continuous variables, means and standard deviations will be presented. In the case of variables with highly skewed distributions, medians, 25th and 75th percentiles will be presented instead. For all variables, the percentage of missing values will be reported. No p-values will be calculated for these tables.

6.4 Analyses for study objectives

The data will be analysed using standard statistical methods such as Generalised Linear Mixed Models (GLMM) and individual-based mathematical modelling of transmission dynamics.

6.5 Statistical analysis of effects of flu vaccination on bacterial transmission

The data will be analysed in two ways within the step wedge design, using both vertical and horizontal analyses. The vertical analysis involves comparisons between two groups randomly allocated to intervention versus no intervention – as this is a true experiment with an intervention, causality can be invoked with much greater confidence to explain any differences observed in rates of transmission as randomisation should control for any confounding effects and biases. The horizontal analysis involves more observations and will have greater power.

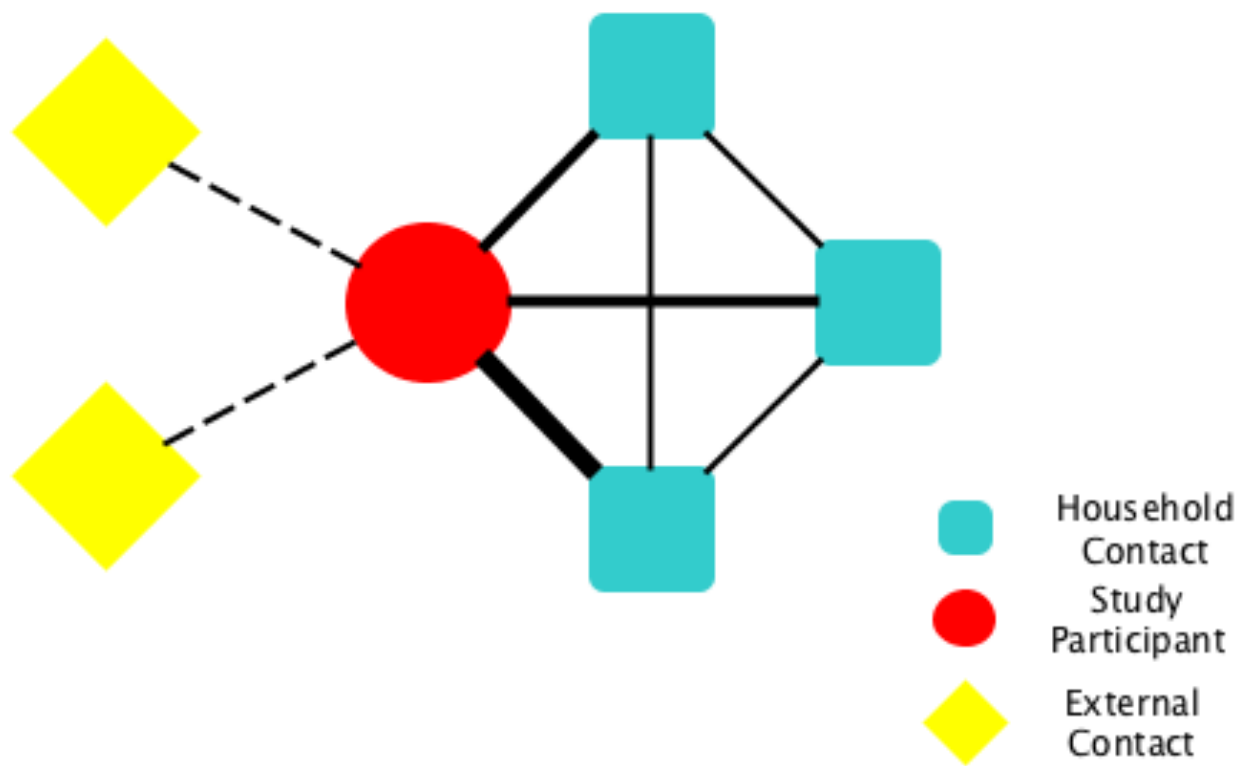

Schematic representing the social contacts of study participants.

The vertical analysis will compare transmission rates in contacts of 2-year-old children randomised to receive LAIV with contacts of children without vaccination at first visit (see definition of transmission in section 3.1). This analysis preserves the randomisation of households. We will treat the transmission rate as the outcome variable, and vaccine status, carriage density, and close-proximity contact duration as key explanatory variables.

The horizontal (longitudinal) analysis will compare pre-immunisation samples (visit 1 in group 1, visits 1-3 in group 2) compared to post-immunisation (visits 2-5 in group 1 and 4-5 in group 2) treating index-case carriage density as a continuous variable and measuring colonisation rates as a function of close-proximity contact duration and carriage density. To account for the clustering of individuals within households and the clustering of repeated measures on individuals, nested hierarchical models will be used for this analysis. In the horizontal models, we will also allow for time trends, due to the possible influence of season on carriage which may confound the effects of the vaccine (for example increases in carriage during the winter). Analyses will be performed in STATA and R.

6.6 Missing data

We will perform statistical analysis on all the individuals and data points available and use multiple imputation to quantify the sensitivity of our analysis to missing data points.

6.7 Mathematical modelling of bacterial transmission dynamics

Beyond statistical analysis of the epidemiological data, we will develop a novel mechanistic mathematical model of bacterial carriage and household transmission. Data from the contact survey will be used to define a network of social contacts, and implement a multi-scale individual-based model. The within-host density in each individual will be modelled as a dynamic variable, dependent on the immunisation status (LAIV+/-) and age category (index child, child contact, adult contact). The probability of onward transmission to each household contact will be modelled as a function of within-host density of the index case, and, crucially, the duration of social contact collected through the contact survey.

The model will be used to characterise the relationship between carriage density and transmission rate, with the exact nature of the relationship to be constrained by the data. The model will be robustly fitted to all study household data simultaneously using state-of-the-art likelihood-free fitting methods, such as Approximate Bayesian Computation. Such an approach will provide estimates of (and uncertainty around) within-host growth rates for vaccinated/unvaccinated index-cases, adults and children, and quantify the functional relationship between carriage density, contact duration and onward transmission. The individual-based model will be implemented in C++ and fitted to study data in R.

7. Direct Access to Source Data / Documents

The CI will allow monitors (from UHB on behalf of the Sponsor), persons responsible for the audit, representatives of the Ethics Committee and of the Regulatory Authorities to have direct access to source data / documents. This is reflected in the Participant Information Sheet (PIS). Study monitoring will be undertaken on behalf of the Sponsor by UHB using their monitoring standard operating procedure (*http://www.uhbristol.nhs.uk/files/nhs-ubht/IS11-Monitoring_v3.5_15.09.2010.pdf*).

7.1 Study Monitoring

7.1.1 Before the Study

The CI will allow the monitor to visit the site and facilities where the study will take place in order to ensure compliance with the protocol requirements.

7.1.2 During the Study

Investigators will allow the monitor and/or the Sponsor to:

- Inspect the site, the facilities and the material used for the study
- Meet all members of his/her team involved in the study
- Consult all of the documents relevant to the study
- Check that the CRFs have been filled out correctly
- Directly access source documents for comparison of data therein with the data in the CRFs (noting that, in this case, the study involves healthy subjects so that clinical data are not recorded in medical records. Accordingly, comparisons will normally be between CRF/data collection forms and computerised data, entered from these forms manually for the purposes of analysis)
- Verify that the study is carried out in compliance with the protocol and local regulatory requirements
- Carry out study monitoring at regular intervals, depending on the recruitment rate, and arranged between the CI and monitor
- All information dealt with during these visits will be treated as strictly confidential

8. Study Conduct and Approvals

The study will be performed subject to all necessary ethics and governance approvals.

This study will recruit children who are not old enough to give consent. Informed consent will be obtained from the parent/legal guardian of each participant

The study will be conducted in accordance with:

- The Medicine for Human Use (Clinical Trial) Regulations 2004.
- International Conference for Harmonisation of Good Clinical Practice (ICH GCP) guidelines.
- Research Governance Framework for Health and Social Care.
- The principles of the Declaration of Helsinki.

9. Data Handling

9.1 Data collection, recording and storage

Data will be collected and retained in accordance with the Data Protection Act 1998.

The study doctors, nurses and administration team will be responsible for data collection, recording and quality control. The study documents (paper and electronic) containing details of demographic data, documentation of inclusion and exclusion criteria, medical and vaccination history will be retained for a period of 15 years following the end of the study. Where trial related information is documented in the medical records, the records will be identified by a ´Do not destroy before dd/mm/yyyy´ label where the date is 15 years after the last patient visit.

Access to these documents would only be available to the investigators, monitors and auditors directly involved in the study. On all study-specific documents, other than the signed consent, the participant will be referred to by the study participant number/initials, not by name.

9.2 Participant Confidentiality

The trial staff will ensure that the participants' anonymity is maintained. The participants will be identified only by initials and a participant number. All documents will be stored securely and will only be accessible to trial staff and authorised personnel. The study will comply with the Data Protection Act which requires data to be anonymised as soon as it is practical to do so. Given the possibility of enrolling twins into this study, the subject record will be labelled with the participant's first name to aid recording of data relevant to the appropriate child.

10. Finance and Insurance

10.1 Finance

The study is financed with collaborative research grant from Pfizer and an unrestricted research grant from the Bill and Melinda Gates Foundation.

10.2 Indemnity and Insurance

The University of Bristol has arranged Public Liability insurance to cover the legal liability of the University as Research Sponsor in the eventuality of harm to a research participant arising from management of the research by the University.

The University of Bristol holds Professional Negligence insurance to cover the legal liability of the University, for harm to participants arising from the design of the research, where the research protocol was designed by the University.

The University of Bristol's Public Liability insurance policy provides an indemnity to our employees for their potential liability for harm to participants during the conduct of the research.

Equivalent appropriate cover will be in place at all study sites.

In addition, investigators have the protection of medical malpractice indemnity with the Medical Protection Society or Medical Defence Union.

11. Publication policy

Presentations of data and publications arising from this study will be coordinated by the chief investigator.

All publications and presentations will acknowledge all sources of financial support. Copies of all publications will be sent to collaborators and funders.

Authorship of publications will include all those who meet all of the following criteria:

i. Substantial contributions to conception and design, acquisition of data, or analysis and interpretation of data.
ii. Drafting the article or revising it critically for important intellectual content.
iii. Final approval of the version to be published.

Other collaborators or members of the research group who may have contributed to some but not all of these criteria will be listed in the Acknowledgments.

Authorship and order of authorship will be agreed in advance of development of publications. Publications will include a description of the contributions of each author and how the order has been assigned. This is likely to place the person who took the lead in writing the manuscript or doing the research first, and the most experienced contributor in the field last.

12. References

1. Thors, V., et al., *Human vaccines & immunotherapeutics* **2016,** *12* (2), 375-82.
2. Finn, A., et al., *The Pediatric infectious disease journal* **2016,** *35* (10), 1080-5.
3. McCullers, J. A., et al., *J Infect Dis* **2010,** *202* (8), 1287-95.
4. Millar, E. V., et al., *The Pediatric infectious disease journal* **2009,** *28* (8), 711-6.
5. Loeb, M., et al., *JAMA* **2010,** *303* (10), 943-50.
6. Collins, A. M., et al., *American journal of respiratory and critical care medicine* **2015,** *192* (7), 853-8.
7. Thors, V., et al., *American journal of respiratory and critical care medicine* **2016,** *193* (12), 1401-9.
8. Seale, A., et al., *Current opinion in infectious diseases* **2011,** *24* (3), 219-24.
9. Weinberger, D. M., et al., *Clin Infect Dis* **2014,** *58* (2), 188-94.
10. Wolter, N., et al., *J Infect Dis* **2014,** *210* (10), 1649-57.
11. Flasche, S., et al., *PLoS medicine* **2015,** *12* (6), e1001839.
12. Rodrigues, F., et al., *The Pediatric infectious disease journal* **2013,** *32*, 227-232.
13. Klugman, K. P., *The Lancet infectious diseases* **2001,** *1* (2), 85-91.
14. Rodrigues, F., et al., *Vaccine* **2012,** *30* (26), 3951-6.
15. Hanage, W. P., et al., *Epidemics* **2010,** *2* (2), 80-4.
16. van Hoek, A. J., et al., *Vaccine* **2014,** *32* (34), 4349-55.
17. Spijkerman, J., et al., *PloS One* **2012,** *7* (6), e39730.
18. O'Brien, K. L., et al., *Journal of clinical microbiology* **2001,** *39* (3), 1021-4.
19. Satzke, C., et al., *PLoS medicine* **2015,** *12* (11), e1001903; discussion e1001903.
20. Rodrigues, F., et al., *PloS One* **2016,** *11* (9), e0163435.
21. Sakai, F., et al., *PloS One* **2015,** *10* (3), e0121064.
22. Bosch, A. A., et al., *PLoS pathogens* **2013,** *9* (1), e1003057.
23. Ruohola, A., et al., *J Infect* **2013,** *66* (3), 247-54.
24. McCullers, J. A., *Nature reviews. Microbiology* **2014,** *12* (4), 252-62.
25. Danon, L., et al., *Proc Biol Sci* **2013,** *280* (1765), 20131037.
26. Hamaluba, M., et al., *Medicine* **2015,** *94* (1), e335.
27. Turner, P., et al., *Journal of clinical microbiology* **2011,** *49* (5), 1784-9.
28. Brugger, S. D., et al., *PloS One* **2010,** *5* (7), e11638.

South West - Cornwall & Plymouth Research Ethics Committee
Level 3
Block B
Whitefriars
Lewins Mead
Bristol
BS1 2NT

<u>Please note: This is the favourable opinion of the REC only and does not allow</u> the amendment to be implemented at NHS sites in England until the outcome of the HRA assessment has been confirmed.

23 July 2018

Prof Adam Finn
David Baum Professor of Paediatrics & Honorary Consultant Paediatrician
University of Bristol
Level 6, Education & Research Centre, Upper Maudlin Street
Bristol
BS2 8AE

Dear Prof Finn

**Study title:**	**Evaluation of the relationship between pneumococcal colonisation density in 2-year-old children and rates of transmission to family contacts using live attenuated intranasal influenza vaccine as a probe. A randomised prospective step-wedge multicentre study.**
**REC reference:**	**17/SW/0190**
**Protocol number:**	**2779**
**Amendment number:**	**1.2**
**Amendment date:**	**08 June 2018**
**IRAS project ID:**	**227810**

The above amendment was reviewed at the meeting of the Sub-Committee held on 30 June 2018 by the Sub-Committee in correspondence.

Summary

1. Addition of further sites to ensure study recruitment target can be met.

2. Changes relating to updated NHS/DoH guidance for LAIV eligibility: upper age limit extended from 9 to 10 years; post-vaccination observation period shortened from 30 to 15 minutes (no observation at all when given routinely).

3. Changes to saliva sample handling, based on staff feedback, as well as minor rewording of some documents to improve clarity.

Ethical opinion

The members of the Committee taking part in the review gave a favourable ethical opinion of the amendment on the basis described in the notice of amendment form and supporting documentation.

Approved documents

The documents reviewed and approved at the meeting were:

Document	*Version*	*Date*
Copies of advertisement materials for research participants [TOP_Poster-Flyer]	1.2	23 May 2018
Covering letter on headed paper		18 June 2018
GP/consultant information sheets or letters [GP letter - enrolment]	1.1	23 May 2018
Letters of invitation to participant	1.1	22 May 2018
Notice of Substantial Amendment (non-CTIMP	2	18 June 2018
Notice of Substantial Amendment (non-CTIMP) [TOP Contact Survey]	1.2	08 June 2018
Other [TOP_instructions - spit sampling]	1.1	22 May 2018
Other [TOP_instructions - nose swab sampling]	1.1	22 May 2018
Other [TOP_Opt-out letter - school-based flu vaccination]	1.2	23 May 2018
Other [TOP_Website text_v1.1_180523_tracked]	1.1	23 May 2018
Participant consent form [TOP_consent form - adult contacts]	1.2	23 May 2018
Participant consent form [TOP_assent form - sibling]	1.1	23 May 2018
Participant consent form [TOP_parental consent form - index child]	1.2	23 May 2018
Participant consent form [TOP_parental consent form - sibling under 16]	1.2	23 May 2018
Participant information sheet (PIS) [TOP_assent info - 6 to 10 years old]	1.2	22 May 2018
Participant information sheet (PIS) [TOP_assent info - 11 to 15 years old]	1.2	22 May 2018
Participant information sheet (PIS) [TOP_assent info - 11 to 15 years old]	1.2	22 May 2018
Participant information sheet (PIS) [TOP_info - adult contacts 16+]	1.2	22 May 2018
Participant information sheet (PIS) [TOP_parent info - siblings less than 16]	1.2	23 May 2018
Participant information sheet (PIS) [TOP_parent info - index child]	1.2	23 May 2018
Research protocol or project proposal [TOP_Protocol]	1.12	24 May 2018

Membership of the Committee

The members of the Committee who took part in the review are listed on the attached sheet.

Working with NHS Care Organisations

Sponsors should ensure that they notify the R&D office for the relevant NHS care organisation of this amendment in line with the terms detailed in the categorisation email issued by the lead nation for the study.

Statement of compliance

The Committee is constituted in accordance with the Governance Arrangements for Research Ethics Committees and complies fully with the Standard Operating Procedures for Research Ethics Committees in the UK.

We are pleased to welcome researchers and R & D staff at our Research Ethics Committee members' training days – see details at http://www.hra.nhs.uk/hra-training/

**17/SW/0190:**	**Please quote this number on all correspondence**

Yours sincerely

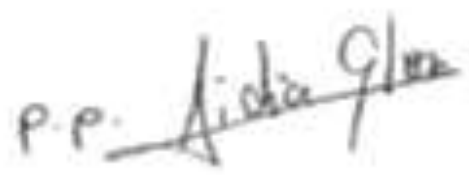

Canon Ian Ainsworth-Smith
Chair

E-mail: nrescommittee.southwest-cornwall-plymouth@nhs.net

Enclosures: *List of names and professions of members who took part in the review*

Copy to: *Jessica Bisset, University Hospitals Bristol NHS Foundation Trust*
Dr Birgit Whitman

South West - Cornwall & Plymouth Research Ethics Committee

Attendance at Sub-Committee of the REC meeting on 30 June 2018

Committee Members:

Name	*Profession*	*Present*	*Notes*
Canon Ian Ainsworth-Smith	Retired Hospital Chaplain	Yes	
Mr Robert Wosley	Deputy Service Line Cluster Manager	Yes	

Also in attendance:

Name	*Position (or reason for attending)*
Miss Charlotte Ferris	REC Manager
